# Predicting distributions of *Wolbachia* strains through host ecological contact—Who's manipulating whom?

**DOI:** 10.1002/ece3.8826

**Published:** 2022-04-13

**Authors:** Clive T. Darwell, Daniel Souto‐Vilarós, Jan Michalek, Sotiria Boutsi, Brus Isua, Mentap Sisol, Thomas Kuyaiva, George Weiblen, Vlastimil Křivan, Vojtech Novotny, Simon T. Segar

**Affiliations:** ^1^ National Center for Genetic Engineering and Biotechnology (BIOTEC), National Science and Technology Development Agency Khlong Luang Thailand; ^2^ Biology Centre Institute of Entomology Czech Academy of Sciences Ceske Budejovice Czech Republic; ^3^ Faculty of Science University of South Bohemia in Ceske Budejovice Ceske Budejovice Czech Republic; ^4^ Agriculture & Environment Department Harper Adams University Newport UK; ^5^ The New Guinea Binatang Research Center Madang Papua New Guinea; ^6^ Department of Plant & Microbial Biology Bell Museum University of Minnesota Saint Paul Minnesota USA

**Keywords:** cytoplasmic incompatibility, fig‐wasp, mutualism, New Guinea, speciation, *Wolbachia*

## Abstract

Reproductive isolation in response to divergent selection is often mediated via third‐party interactions. Under these conditions, speciation is inextricably linked to ecological context. We present a novel framework for understanding arthropod speciation as mediated by *Wolbachia*, a microbial endosymbiont capable of causing host cytoplasmic incompatibility (CI). We predict that sympatric host sister‐species harbor paraphyletic *Wolbachia* strains that provide CI, while well‐defined congeners in ecological contact and recently diverged noninteracting congeners are uninfected due to *Wolbachia* redundancy. We argue that *Wolbachia* provides an adaptive advantage when coupled with reduced hybrid fitness, facilitating assortative mating between co‐occurring divergent phenotypes—the *contact contingency* hypothesis. To test this, we applied a predictive algorithm to empirical pollinating fig wasp data, achieving up to 91.60% accuracy. We further postulate that observed temporal decay of *Wolbachia* incidence results from adaptive host purging—*adaptive decay* hypothesis—but implementation failed to predict systematic patterns. We then account for post‐zygotic offspring mortality during CI mating, modeling fitness clines across developmental resources—the *fecundity trade*‐*off* hypothesis. This model regularly favored CI despite fecundity losses. We demonstrate that a rules‐based algorithm accurately predicts *Wolbachia* infection status. This has implications among other systems where closely related sympatric species encounter adaptive disadvantage through hybridization.

## Graphical abstract

It is often considered that a large proportion of arthropod diversity harbors a non‐systematically distributed agent of speciation (*Wolbachia*). We present a novel framework for studying arthropod speciation as mediated by *Wolbachia* under a paradigm of host‐adaptive response contingent on ecological circumstances. Our results indicate that *Wolbachia* distributions are systematically structured among an arthropod dataset.

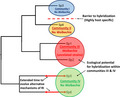

## 1 INTRODUCTION

Recognizing the conditions that favor speciation is critical if we are to understand the extent and structure of biodiversity. Species interactions, both between and within trophic levels, can be significant contributors to diversification processes (Segar et al., 2020). These are sculpted by evolutionary forces, which in combination with abiotic drivers deliver an ecosystem or community's (dynamic) state (Harmon et al., 2019). Thus, an in‐depth understanding of adaptive processes alongside their ecological contingencies (Segar et al., 2020) is fundamental to achieving standard objectives in ecology.

Apropos of this, the arthropod microbiome, as a modifier of ecological interaction strength, is increasingly viewed as a critical factor (e.g., Hansen & Moran, 2014). One such endosymbiotic bacterium, *Wolbachia*, infects around half of arthropod species (Weinert et al., 2015) often playing a key role in speciation (Bordenstein et al., 2001; Jaenike et al., 2006; Nice et al., 2009; Shropshire & Bordenstein, 2016; Telschow et al., 2007). *Wolbachia* commonly induces cytoplasmic incompatibility (CI) via sexual sterility between infected males and females that are either uninfected (*unidirectional*‐*CI*) or carry an alternative strain (*bidirectional*‐*CI*) (Beckmann et al., 2017, 2019; LePage et al., 2017). CI may therefore promote reproductive isolation (RI) between populations or incipient host species and increase the speed or likelihood of speciation by restricting geneflow (Bordenstein et al., 2001; Telschow et al., 2005, 2007; Zimmer, 2001).

Among arthropods, *Wolbachia* lineages are mostly facultative and evolutionarily unstable symbionts generally exhibiting host co‐phylogenetic incongruence (Jäckel et al., 2013; Shoemaker et al., 2002; Yang et al., 2012), although exceptions are known where essential mutualism appears likely (Dedeine et al., 2001; Hamm et al., 2014; Raychoudhury et al., 2009). *Wolbachia* often appears idiosyncratically distributed among closely related hosts that harbor paraphyletic strains (Jäckel et al., 2013; Shoemaker et al., 2002; Smith et al., 2012; Yang et al., 2012). Horizontal exchange may occur between unrelated species (Bailly‐Bechet et al., 2017; Shoemaker et al., 2002; Zug et al., 2012). Counterintuitively, this may not be readily predicted from close ecological contact (Gerth et al., 2013; Haine & Cook, 2005; Jäckel et al., 2013), but identified incidences (McFrederick & Rehan, 2016; Miraldo & Duplouy, 2019; Sintupachee et al., 2006) suggest that outcomes may be context dependant. While *Wolbachia* may confer host benefits (Teixeira et al., 2008), a consensus view is that *Wolbachia* represents a net cost meaning its infection status typically depends on its ability to manipulate its host (Werren, 2011).

*Wolbachia* is posited to facilitate reproductive isolation between incipient species in combination with reduced hybrid fitness (Shoemaker et al., 1999). The maladaptation of intermediate hybrid forms is central to models of sympatric/ecological speciation (Rundle & Nosil, 2005), and an alternative view from convention may treat CI as a net benefit rendering divergent host fitness advantages (i.e., via hybrid avoidance). If so, selection on hosts would be the prime determinant of infection status, rather than the bacterium's manipulative capability. However, as CI results in post‐zygotic mortality, fitness losses imply that a balancing counter mechanism must also operate (*sensu* Caspari & Watson, 1959).

Predictive phylogenetic approaches to understanding *Wolbachia* distributions have not previously incorporated ecological contact between insect lineages as a discriminant factor regulating CI. Moreover, attempts have focused solely on host systematic patterns (Engelstädter & Hurst, 2006a) without incorporating either abiotic or biotic (e.g., community network) drivers. When allopatric speciation occurs, specific mechanisms of RI may not necessarily evolve as nascent species are not in contact (Coyne & Orr, 2004). This may also be true if newly formed species specialize on different resources in sympatry (Nosil, 2012). However, RI is required if ecological contact occurs during critical periods (e.g., mating windows) (Via & Hawthorne, 2002)—hereafter termed the *contact contingency* hypothesis. *Wolbachia* typically suffers drop out from host lineages (Bailly‐Bechet et al., 2017; Koehncke et al., 2009), exacerbating host–symbiont phylogenetic incongruence and invoking the idea that eventual purging by hosts may occur. Compared with *Wolbachia*, alternative mechanisms of RI requiring cytogenetic or morphological modification may take longer to evolve (Bordenstein et al., 2001; Coyne & Orr, 2004) and be relatively unresponsive to changing circumstances favoring diversification. Thus, *Wolbachia* purging could result from temporal changes in its relative adaptive benefits (as alternative mechanisms of RI evolve) that subsequently become redundant and eradicated if selection acts on host mutations (Koehncke et al., 2009), potentially via immune responses—hereafter termed the *adaptive decay* hypothesis.

In general, the view that a large proportion of arthropod diversity (Weinert et al., 2015) harbors a non‐systematically distributed agent of speciation constitutes a major academic challenge when identifying unifying processes underpinning biodiversity. Failure to comprehensively evaluate whether arthropod diversification regularly occurs stochastically entails circumvention of endeavor in attempting to fully unpick the eco‐evolutionary and biogeographic histories of the planet's most diverse phylum—a position that significantly resonates across the key debate surrounding the relative contributions of adaptive (Chase & Leibold, 2003; Chesson, 2000) versus neutral (Hubbel, 2001) process in structuring biodiversity. Thus, attempts to solve it are essential even if they solely establish that the investigated dynamics are indeed unpredictable. While demonstration of predictable patterns would enhance the debate around whether CI drives speciation or is merely subordinately associated with it (Bruzzese et al., 2021).

In pollinating and non‐pollinating fig wasps (Chalcidoidea), where *Wolbachia* prevalence is ca. 60%, many closely related species share enclosed reproductive spaces (i.e., fig syconia), meaning regular contact and potential for hybridization (Darwell et al., 2014; Molbo et al., 2003; Yu et al., 2019). Inbreeding is common, favoring female‐biased sex ratios promoting geneflow barriers and endosymbiont strain fidelity (Branca et al., 2009). The confined nature of syconia means incipient speciation would require rapidly developed RI (Nosil, 2012) to avoid hybridization costs. Fig wasps regularly show paraphyletic *Wolbachia* infections across sister‐species (Haine & Cook, 2005; Shoemaker et al., 2002; Yang et al., 2012), while species occupying communities without congeners invariably display null *Wolbachia* statuses (Haine & Cook, 2005).

**FIGURE 1:**
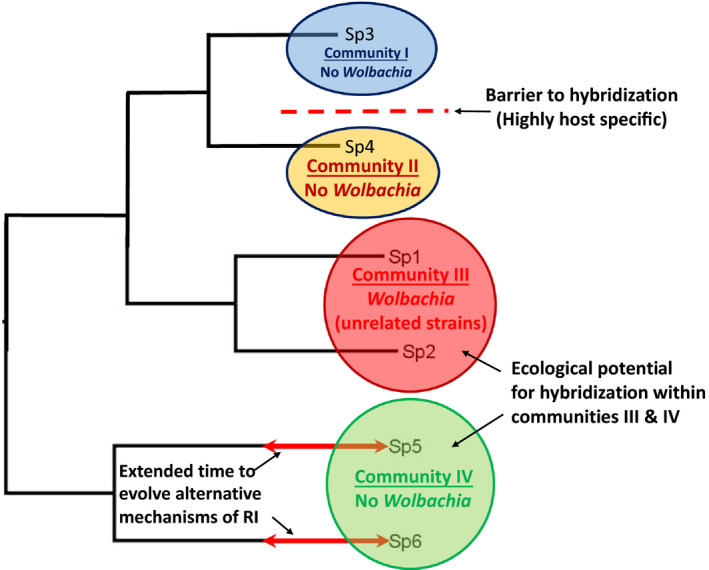
Conceptual diagram outlining the “contact contingency” hypothesis. Hypothetical fig wasp relationships and predicted status of RI inducing *Wolbachia* according to variation in ecological contact and evolutionary time since speciation. We predict *Wolbachia* infection to occur only in community III where species 1 and 2 should harbor unrelated strains. Sister species 3 and 4 are not in ecological contact as they form separate communities I and II, while sister‐species 5 and 6 in community IV, despite ecological contact, have had sufficient evolutionary time for alternative (less costly) RI mechanisms to evolve. Our framework represents a predictive framework that nevertheless elicits an apparently stochastic distribution, as is frequently observed

Hybridization is costly between highly adapted lineages of fig wasps, featuring narrow abiotic niches (Darwell et al., 2014) and extreme matching for host fig interacting traits (Weiblen, 2004), which tend to form well‐defined species (Souto‐Vilarós et al., 2019). We develop a framework which favors ecologically contingent host tolerance of otherwise costly *Wolbachia*, whereby incipient species are infected with paraphyletic *Wolbachia* when in ecological contact (“contact contingency”; Figure 1). Thus, *Wolbachia* facilitates adaptive divergence while selecting for less‐costly pre‐zygotic mechanisms (Telschow et al., 2005, 2007) to subsequently evolve which we model as purging of *Wolbachia* after extended timescales (“adaptive decay”). Thus, we emphasize niche‐based adaptive processes among hosts (e.g., Cody et al., 1975), over neutral dynamics that may be invoked if considering *Wolbachia* invasion a more stochastic phenomenon.

**FIGURE 2:**
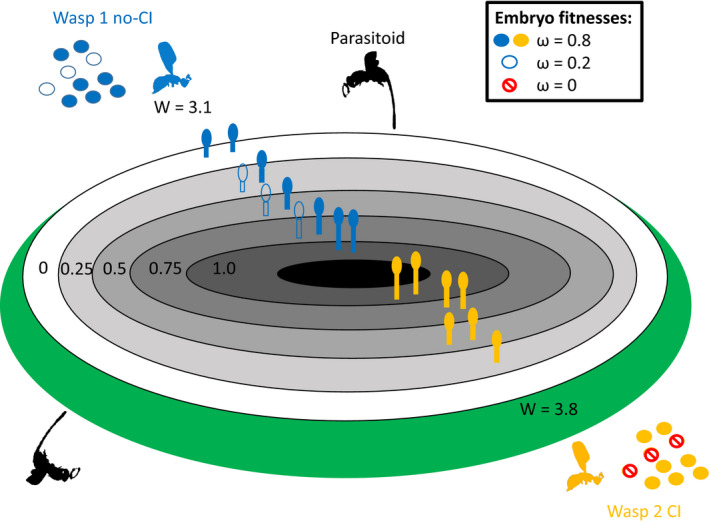
Stylized schematic showing a fig in cross section. Five layers of ovules are used in our “fecundity trade‐off” model (white and grey) and no oviposition occurs in the central lumen (black). Ovule length (and embryo relative fitness, ω) decreases towards the fig wall (green) where larvae are at greater risk of parasitism. We use a descriptive model to contrast inclusive fitness (W) between foundress wasps that do not experience cytoplasmic incompatibility (wasp 1, blue) and those that do (wasp 2, orange). Here, in a toy example, each foundress has 10 eggs (open circles represent viable hybrid eggs with decreased fitness while closed circles are non‐hybrids with full fitness) and we limit oviposition to two eggs per layer. While CI wasps lay fewer eggs (hybrids are lost to CI) they do not fill valuable oviposition sites with hybrids of decreased fitness. Here, the CI wasp gets an inclusive fitness of 3.8 for its seven remaining eggs and the noninfected wasp gets 3.1 for a full complement of 10 eggs (i.e., by multiplying egg fitness by oviposition fitness then summing). Inclusive fitness is therefore greater in wasp 2 despite this fecundity loss, as it lays a higher number of high fitness eggs in premium oviposition sites. This example would represent one pixel on the heat maps displayed in Figure 7. Please see text for further details

As noted, our framework requires an auxiliary mechanism tolerant of post‐zygotic fecundity reduction. We consider conditions for species where offspring experience heightened competition for developmental resources. Oviposition sites are finite for pollinating fig wasps unable to exit syconia after entry (Cook & Segar, 2010). Central sites are increasingly valuable as parasitoid wasp ovipositors typically do not penetrate zygotes there (e.g., Dunn et al., 2008). Post‐zygotic fecundity reduction may prove tolerable if hybrid eggs do not waste premium sites (or other resources; hereafter termed the *fecundity trade*‐*off* hypothesis). This could feasibly occur in fig wasps via: (i) preferential oviposition of favored non‐hybrid embryos (Hymenoptera at least have documented oviposition preference according to ploidy; Raja et al., 2008) in central syconia layers; or (ii) via differential mortality affecting unviable hybrids before oviposition (suggested for Drosophila CI; Weeks et al., 2007). This assumes that multiple mating events occur within syconia (Greeff et al., 2003; Murray, 1990). We model this by simulating pre‐oviposition egg mortality causing reduced egg load (zero fitness for lost hybrid embryos) resulting in the favorable oviposition of higher fitness eggs (Figure 2).

We tested our “contact contingency” and “adaptive decay” hypotheses on several monophyletic fig (*Ficus*, Moraceae) species complexes and their pollinating wasps along a steep elevational gradient featuring clinal turnover of *Ficus* species in Papua New Guinea (Segar et al., 2017). After screening wasps for *Wolbachia*, we simulated our proposed mechanisms across the empirical dataset incorporating ecological contact and phylogenetic relationships before evaluating predictive accuracy. For our “fecundity trade‐off” hypothesis, we present a full functional model for evaluation. Finally, we use RAD‐seq data to evaluate whether CI likely operates in our system.

## 2 METHODS

### 2.1 Field collection

We collected pollinating fig wasps from one species complex (*Ficus itoana*, *F*. *umbrae*, and *F*. *microdyctia*) two sub‐species (*F*. *trichocerasa* subsp. *trichocerasa* and subsp. *pleioclada*) and two species with wide elevational ranges (*F*. *wassa* and *F*. *arfakensis*). Syconia from 10–15 fig individuals at each location were placed into breathable rearing pots until pollinating wasps emerged. Five male and five female wasps from each syconia were stored in 2‐ml tubes with silica gel and a small piece of cotton wool before long‐term storage at −20°C (Moe & Weiblen, 2012).

### 2.2 DNA extraction and sequencing

All wasp materials, DNA extraction, and RADseq protocols follow Souto‐Vilarós et al. (2019). We used primers and protocols from Baldo et al. (2006) to amplify *Wolbachia* surface protein gene (*wsp*) and five Multi Locus Strain Typing (MLST) genes used for strain typing. Alignment was conducted in BioEdit (Hall, 1999), while sequencing was conducted at Macrogen Europe.

### 2.3 Strain typing and phylogenetic inference

All *wsp* and MLST sequences were compared to the MLST data base (Jolley et al., 2018) to assess strain similarity. Final strain delimitation was based on: (i) consistency of allele assignation across MLST loci and *wsp* and (ii), phylogenetic evidence. Separate phylogenies were generated for all genes using RAxML v8.2.12 (default settings, GTRGAMMA with no invariant sites) to assess consistency of strain groupings across genes and verify that no two strains formed a monophyletic group across the MLST data base. A non‐partitioned multi‐gene phylogeny was estimated using ExaBayes v.1.4.1 (default settings for one run, majority rules consensus using a threshold of 50%) and rooted on *Wolbachia* sequences associated with a species of *Pleistodontes* pollinating wasp collected at Mt Wilhelm. The phylogeny of pollinating wasps was estimated using genomic data taken from Souto‐Vilarós et al. (2019) using ExaBayes (Aberer et al., 2014) as outlined above. As discussed by Baldo et al. (2006), the MLST approach is more robust than a purely phylogenetic approach because recombination is frequent in *Wolbachia*. We follow Baldo et al. (2006) and classify strains identical at three or more alleles as close relatives, using phylogenetic distance as a secondary source of evidence.

### 2.4 A framework for the “Contact contingency” and “adaptive decay” hypotheses

First, we evaluated how different hypotheses of host wasp species diversity would influence the predictive abilities of our contact contingency hypothesis. For this, we considered multiple assessments of putative species (i.e., species delimitation hypotheses) as characterized by their elevational distribution breath (expressed in elevations where they occur) and their association with one of six host fig species. For example, let us consider four elevation sites *i* = 1,2,3,4, at elevations 200, 700, 1200, and 1700 m.a.s.l. We define a putative species as a quadruple *e*_1_ *e*_2_ *e*_3_ *e*_4_ where *e_i_* is either equal to 0 when the species does not occupy an elevation site *i*(= 1,2,3,4) or 1, if it does. We assume that a putative species may only belong to a single fig host because real fig wasps are almost always host specific. We assume elevations are sorted in increasing order, so that elevation site 1 is lower than elevation site 2 etc. Thus, a putative species that occupies the first three elevation sites is 1110. We further assume that the elevation range of a putative species is continuous—for example, 1101 is not considered as a putative species, because there is a gap in its distributional breath. For four elevation sites, there are 10 distinct putative species 1111, 1110, 0111, 1100, 0110, 0011, 1000, 0100, 0010, and 0001. Based on empirical network structure where any two wasp species do not overlap at one or more elevations, we assume that putative species also do not overlap at any single elevation site. For example, putative species 1110 and 0010 cannot be associated with a fig host as they overlap at elevation site *i* = 3. This assumption reduces the number of all possible combinations of putative species within a fig host. For example, with four elevation sites, there is a single combination {1111} of a single putative species that occupies all elevation sites. There are three possible combinations of two putative species {1000,0111},{1100,0011},{1110,0001}, three combinations of three putative species {1000,0100,0011},{1100,0010,0001},{1000,0110,0001}, and a single combination of four putative species {1000,0100,0010,0001}. There are 2^3^ = 8 combinations of putative species in this example. In general, if a host fig is distributed across *n* elevation sites, there are then 2*^n^* ^−1^ possible putative species combinations.^1^ From empirical data, we know that populations of wasps within *F*. *arfakensis* are distributed across 4 elevations, *F*. *umbrae* across 1 elevation, *F*. *itoana* across 2 elevations, *F*. *microdictya* across 2 elevations, *F*. *trichocerasa* across 4 elevations, and *F*. *wassa* across 6 elevations. Thus, the number of all possible combinations of putative species among these six fig hosts are *n_a_* = 8, *n_u_* = 1, *n_i_* = 2, *n_m_* = 2, *n_t_* = 8, and *n_w_* = 32, where subindex denotes the respective community (*a*‐*F*. *arfakensis*, *u*‐*F*. *umbrae*, *i*‐*F*. *itoana*, *mF*. *microdictya*. *t*‐*F*. *trichocerasa*, *w*‐*F*. *wassa*). Multiplying these numbers, we obtain 8192 possible putative species combinations (hereafter PSCs) across the six fig species.

### 2.5 Calculating phylogenetic distances between putative species

**FIGURE 3:**
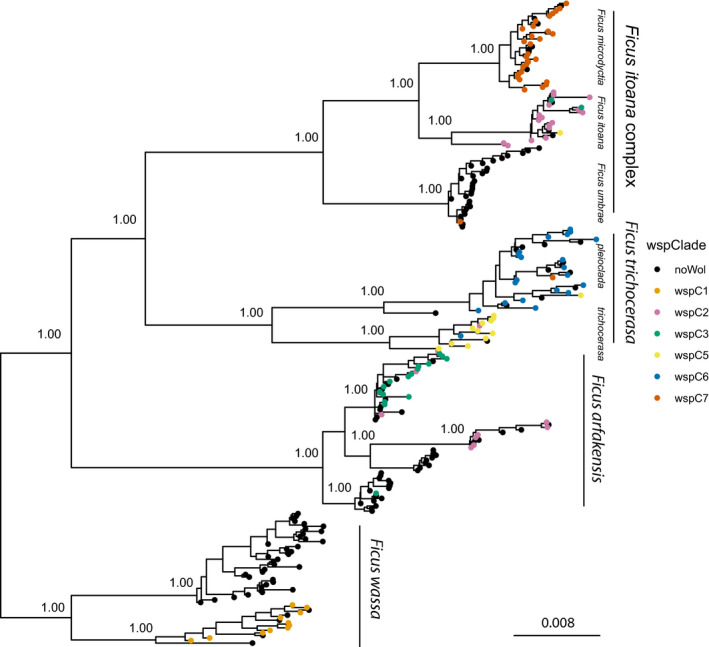
*Wolbachia* strains mapped along the pollinating wasp phylogeny. Strain type is indicated by the different colors, with uninfected individuals in black. For each wasp clade the *Ficus* community is given. Node labels give Bayesian posterior probability support. The tree was rooted to *Wolbachia* extracted from a species of *Pleistodontes* pollinating fig wasp sampled MtWilhelm. Scale bar in substitutions per site

Next, we calculate phylogenetic distances among putative species within each PSC. For example, there are 64 *F*. *arfakensis* individuals (Figure 3) each associated with one of the four elevations (i.e., 200, 700, 1200, 1700 m.a.s.l.) where each was collected; thus, there are 2^3^ = 8 possible combinations of putative species. For example, one such combination comprising three putative species is {psp_1_,psp_2_,psp_3_} = {1000,0100,0011}. Thus, all observed individuals at elevation site 200 m.a.s.l. are grouped representing putative species psp_1_, all individuals observed at elevation 700 m.a.s.l. represent psp_2_, and all individuals collected at elevation sites 1200 and 1700 m.a.s.l. represent psp_3_. Such grouping of individuals allows us to calculate phylogenetic distances among these three putative species using the real phylogenetic tree.

The minimum phylogenetic distance between the two nearest individuals among all pairs of putative species is taken. For the *F*. *arfakensis* example, this results in three pairwise distances between the three putative species. For example, if there are 5 wasps in putative species psp_1_ and 3 wasps in psp_2_, then we calculate phylogenetic distances between any two individuals $d\left(T_{i}^{{\text{psp}}_{1}},T_{j}^{{\text{psp}}_{2}}\right)$ where $T_{i}^{{\text{psp}}_{1}}$, *i* = 1,…,5 denote individuals belonging to putative species psp_1_ and $T_{j}^{{\text{psp}}_{2}}$, *j* = 1,2,3 denote individuals belonging to putative species psp_2_. In this case there will be 15 such distances between individuals and to calculate the phylogenetic distance between putative species psp_1_ and psp_2_, we take minimum of these distances, that is,

$$d\left({\text{psp}}_{1},{\text{psp}}_{2}\right)=\min_{i=1,\ldots ,5;j=1,\ldots ,3}d\left(T_{i}^{{\text{psp}}_{1}},T_{j}^{{\text{psp}}_{2}}\right)$$

### 2.6 Ascribing Wolbachia infections

We assume that when there are at least two putative species in a host fig community then each of them is infected with a different *Wolbachia* strain. If there is only a single putative species in a community, we do not associate any *Wolbachia* strain with it. We assume wasp species are host‐specific at the host species level but that *Wolbachia* still persists in the “itoana” species complex (*F*. *umbrae*, *F*. *microdictya*, and *F*. *itoana*) as it is a recent speciation event—and therefore still treated as a community, although wasps may make “mistakes” about their host due to conservative evolution of host fig chemical attractants, especially if they do not have the “correct” host as an option. Thus, our four communities are “arfakensis,” “wassa,” “trichocerasa,” and “itoana” (species complex). For example, for the combination of putative species {psp_1_,psp_2_,psp_3_}, we assign three different *Wolbachia* strains (denoted *w*_1_−*w*_3_) to each of these putative species, that is, {psp_1_ *^w^* ^1^, psp_2_ *^w^* ^2^, psp_3_ *^w^* ^3^}.

If any inter‐putative species distances are above the evaluated purge threshold, then all individuals among them have their *Wolbachia* assignations removed; however, this is only done providing that the distance between any other third‐party putative species is not below the evaluated threshold. For example, in our *F*. *arfakensis* example, if the evaluated purge threshold is 0.05, and pairwise distances between putative species are: *d*(psp_1_,psp_2_) = 0.03; *d*(psp_1_,psp_3_) = 0.06; and *d*(psp_2_,psp_3_) = 0.06, then only *Wolbachia* assignation attributed to psp_3_ is removed as psp_1_ and psp_2_ are separated by a distance less than the evaluated threshold, that is, purging is done conservatively at each evaluated purge threshold (ranging incrementally from zero—that is, no purging—to the maximum pairwise distance recorded in the phylogeny, and performed across all putative species combinations). This assumption corresponds to our adaptive decay hypothesis that CI is obsolete for reproductive isolation if two putative species are evolutionarily distant. We observe, there are just two possibilities for each putative species’ simulated infections among multiple species communities: either all individuals retain their single infection status, or all individuals have no *Wolbachia* strain.

After the purging step is complete, we calculate the *Wolbachia* infection accuracy (hereafter WIA) score between simulated and empirical *Wolbachia* strains. For example, assume we have six wasps (*T*_1_−*T*_6_) belonging to two putative species each ascribed distinct *Wolbachia* strains: psp_1_ *^w^* ^1^ and psp_2_ *^w^* ^2^ (Table 1ai and bi). Further assume these individuals are infected by three *Wolbachia* strains W1‐W3 as shown. To calculate the accuracy score, we define a matrix where empirical *Wolbachia* strain infections are in columns and predicted putative species infections are in rows and the entries calculate the number of correctly predicted individuals for the particular combination of ascribed and real *Wolbachia* strains.

**TABLE 1:** (ai and bi) Two tables each showing sortings of six wasps belonging to two putative species psp_1_ and psp_2_ with two ascribed *Wolbachia* strains (*w1* and *w2*). These individuals were typed as having three real recorded *Wolbachia* strains (W1, W2 and W3). (aii and bii) The corresponding matrices that evaluate fit between real species and putative species. (aii) the accuracy score is 2 + 2 = 4. For bii there is a clash in sorting, because the row maxima are achieved in the same column (i.e., from the same empirical *Wolbachia* strain). To avoid such a clash, we replace the bottom row value with the next highest value (the record for *w2*‐W2 is struck out) from a column where the empirical strain has not been recorded from another row: 2 + 1 = 3

(ai)			
Wasp	Putative species	Ascribed *Wolbachia* infection	Real *Wolbachia* infection
*T*_1_	psp_1_	*w1*	W1
*T*_2_	psp_1_	*w1*	W1
*T*_3_	psp_1_	*w1*	W2
*T*_4_	psp_2_	*w2*	W3
*T*_5_	psp_2_	*w2*	W3
*T*_6_	psp_2_	*w2*	W2

(aii)				
	W1	W2	W3	Max
*w1*	2	1	0	2
*w2*	0	1	2	2
WIA				4

(bi)			
Wasp	Putative Species	Ascribed *Wolbachia* infection	Real *Wolbachia* infection
*T*_1_	psp_1_	*w1*	W1
*T*_2_	psp_1_	*w1*	W2
*T*_3_	psp_1_	*w1*	W2
*T*_4_	psp_2_	*w2*	W2
*T*_5_	psp_2_	*w2*	W2
*T*_6_	psp_2_	*w2*	W3

(bii)				
	W1	W2	W3	Max
*w1*	1	2	0	2
*w2*	0	2	1	1
WIA				3

Abbreviation: WIA, *Wolbachia* infection accuracy.

The accuracy matrices corresponding to two examples are shown in Table 1aii and bii. In example one, the fit between ascribed *Wolbachia* strain *w1* and real strain W1 equals 2 (Table 1aii). Thus, we observe that the sum of entries in each row of the matrix equals the number of individuals ascribed each individual *Wolbachia* strain. We sum the maximum values in each row to calculate the accuracy score = 4 (Table 1aii). This guarantees that if there is a perfect fit between real *Wolbachia* infections and ascribed *Wolbachia* infections to putative species, then the score is maximum possible and equals to the number of collected individuals.

However, it may happen that row maxima for two or more ascribed *Wolbachia* infections occur within the same column. This is the case for the matrix in Table 1bii. Here, there is a “clash” because the two ascribed *Wolbachia* strains are maximally associated with a single real strain (i.e., W2). In such a case, we calculate the highest sum of row maxima providing a single column is only represented once at most across rows, moving to one of the next highest values from unused columns if the maximum value is not available due to clashes. Thus, each row and each column are only used a maximum of once each when calculating the *Wolbachia* infection accuracy score. The accuracy score for the case in example Table 1b is equal to 3. Finally, uninfected *Wolbachia* infection accuracy (uWIA) is more easily assessed comparing predicted versus empirical typing data.

### 2.7 Implementation

We computationally simulated the above methods outlining the contact contingency and adaptive decay hypotheses using python3 (https://github.com/ctdarwell/wolPredictor). The main program is called wolpredictor which performs all calculations for the contact contingency and adaptive decay (including instances where adaptive decay is not performed) hypotheses including evaluation of *Wolbachia* infection accuracy. The project contains other useful scripts to replicate our analyses. Hereafter, we refer to wolpredictor to describe the full implementation of these approaches and their outputted metrics. As an additional resource alongside our mathematical formalization, a schematic description of its operation is outlined in Figure S1.

### 2.8 Statistical evaluation

To measure how a particular combination of putative species fits the real data, we introduce *putative species combination* (PSC) *accuracy score*. For each putative species combination, the score evaluates the number of correctly assigned wasp individuals across putative species relative to corresponding species derived from the empirical species assessment from our wasp phylogeny (using the *taxdegMatcher.py* script and following species diversity assessment among these fig hosts from Souto‐Vilarós et al., 2019). The method for calculating this metric is identical to calculating the accuracy score between simulated and empirical *Wolbachia* strains (above). An illustrated example is given in Table 2. Finally, we list the assumptions of these frameworks in Table 3.

**TABLE 2:** Tables showing calculation of putative species combination (PSC) accuracy score. (a) frequencies of real species designations and putative species assignments to six individuals within a single community; (b) matrix to evaluate putative species combination (PSC) accuracy score

(a) Wasp	Real Species	Putative species
*T*1	arf1	psp1
*T*2	arf1	psp1
*T*3	arf1	psp2
*T*4	arf2	psp3
*T*5	arf2	psp3
*T*6	arf2	psp1

(b)				
	psp1	psp2	psp3	Max
arf1	2	1	0	2
arf2	1	0	2	2
PSC score				4

**TABLE 3:** Assumptions of the simulation approach

The number of elevation sites for community *F*. *arfakensis*	4
The number of elevation sites for community *F*. *umbrae*	1
The number of elevation sites for community *F*. *itoana*	2
The number of elevation sites for community *F*. *microdictya*	2
The number of elevation sites for community *F*. *trichocerasa*	4
The number of elevation sites for community *F*. *wassa*	6
Putative species in the same community have non‐overlapping elevations	NA
Putative species in different communities are different	NA
Putative species in a community have different *Wolbachia* strains	
A community containing a single species is *Wolbachia* free	

Predictive accuracy was then assessed by regression analyses of PSC accuracy against *Wolbachia* infection accuracy (WIA; cube‐transformed following residual plot evaluations). We also performed regression analyses evaluating whether our inclusion of adaptive decay improved wolpredictor performance (i.e., uWIA) over initial predictions under contact contingency and improved uWIA where positive WIA is not compromised. Finally, we evaluated *Wolbachia* infection accuracy performance against the empirical species richness assessment (Souto‐Vilarós et al., 2019), and, as null comparisons, against an equivalent dataset (8192 combinations) featuring randomized species cluster associations.

### 2.9 “Fecundity trade‐off” hypothesis

Our “fecundity trade‐off” hypothesis assumes an average female lays *N* eggs fertilized by conspecific (*N*_c_) or by heterospecific males (*N*_h_, *N* = *N*_c_ + *N*_h_). Proportions of these are $\pi_{c}=N_{c}/N,\;\pi_{h}=N_{h}/N$. The probability an egg survives to adulthood for conspecific (heterospecific) mating is *ω*_c_ (*ω*_h_). Survival depends on egg parasitization risk according to oviposition across patches; here, the central patch A and the boundary patch B. Survival probabilities are *ω*_A_ and *ω*_B_ (*ω*_A_ > *ω*_B_). Females preferentially oviposit in patch A and subsequently patch B when A is full (Jousselin et al., 2001).

#### 2.9.1 Non‐preferential oviposition

Under “*no CI*,” a female randomly oviposits conspecific and heterospecific eggs. We assume the maximum number of eggs that can be oviposited in the central (boundary) layer A (B) is *n*_A_ (*n*_B_). Assuming that the number of oviposition sites is larger than is the number of eggs a female lays (*n*_A_ + *n*_B_ > *N *> *n*_A_), the number of conspecific (heterospecific) eggs oviposited in patch A is *N*_c_/*N n*_A_ (*N*_h_/*N n*_A_). Remaining eggs are oviposited in patch B, so the number of conspecific (heterospecific) eggs oviposited there is *N*_c_ − *N*_c_/*Nn*_a_ (*N*_h_ − *N*_h_/*Nn*_a_). Let *ω*_A_ (*ω*_B_) be survival probability of eggs oviposited in patch A (B). So, fitness under random egg oviposition is:

$$W_{r}=\omega_{c}\frac{N_{c}}{N}n_{A}\omega_{A}+\omega_{c}\left(N_{c}‐\frac{N_{c}}{N}n_{A}\right)\omega_{B}+\omega_{h}\frac{N_{h}}{N}n_{A}\omega_{A}+\omega_{h}\left(N_{h}‐\frac{N_{h}}{N}n_{A}\right)\omega_{B}.$$

#### 2.9.2 Preferential oviposition

The key assumption under “*CI*” is that unviable eggs are not oviposited (e.g., via heterospecific egg degradation) and thus do not waste premium oviposition sites (equivalent to prioritization of egg oviposition order), permitting preferential oviposition of conspecific offspring. Heterospecific eggs have zero fitness under CI, that is, *ω*_h_ = 0 in formula for *W*_r_. There are two possibilities: (1) If all conspecific eggs are oviposited in the central patch A (i.e., *N*_c_ < *n*_A_) fitness is *W*_p_ = *ω*_C_ *N*_C_ *ω*_A_; we observe that, trivially, *W*_p_ > *W*_r_ as we assume *ω*_A_ > *ω*_B_ and *n*_A_ < N. (2) Not all conspecific eggs are oviposited in the central patch A (i.e., *N*_c_ > *n*_A_). In this case, only *n*_A_ conspecific eggs are oviposited in the central patch and *N*_c_ − *n*_A_ conspecific eggs are oviposited in patch B. Fitness is then *W*_p_ = *ω*_c_ *n*_A_ *ω*_A_ + *ω*_c_(*N*_c_ − *n*_A_) *ω*_B_. Once again, we see that *W*_p_ > *W*_r_ as *N*_c_ < *N*. We use python3 (https://github.com/ctdarwell/wolPredictor/blob/master/ciFitnessModel.py) to computationally simulate this approach. To better approximate clinal oviposition fitness due to increasing risk of parasitism, we consider five oviposition layers (with fitness coefficients of 1, 0.75, 0.5, 0.25 and 0 at each layer). These values correspond to probabilities derived from empirical studies (Dunn et al., 2008). See github pages for further details of implementation.

### 2.10 Evidence for cytoplasmic incompatibility

We evaluated empirical evidence for CI in our system by examining the distribution of cytoplasmic incompatibility factor (*cifA* and *cifB*) genes (Shropshire et al., 2021). We used the basic local alignment search tool (blastn) to identify *cif* genes within our raw wasp next‐RAD reads. We compiled a reference sequence query from *Ceratosolen*, four species of *Drosophila*, and *Wolbachia pipientis* to identify hits (Table S1). We filtered the raw data to include only reads ≥80 base pairs length and with ≥3 copies (across populations) and translated reads to assess functionality (Lindsey et al., 2018) using a custom python3 script (the reference, C. solmsi—GenBank QTP63507, is highly likely to cause CI; Xiao et al., 2012).

## 3 RESULTS

### 3.1 Wolbachia screening of field collected samples

Phylogenetic analyses indicate empirical species richness of 11 pollinating fig wasp species (Figure 3). From 253 screened wasps, 47.0% (119 individuals) carry *Wolbachia* infections. Individual *wsp* and MLST phylogenies confirm the disjunct monophyly of identified strain clades (Table S2; Figures S2 and S3); the *wsp* tree contained five major clades while the MLST tree contained six. *wsp* clade assignment mostly matched MLST markers, except for distinct MLST clades for *F*. *trichocerasa* subsp. *pleioclada* and subsp. *trichocerasa*. We followed *wsp* clade designations with *wsp* clade six split into two (wspC6 and wspC7) giving six identified *Wolbachia* clades. None of our strain types are closely related strains according to Baldo et al. (2006) (e.g., none share three or more alleles).

Three of the six host fig species carry multiple *Wolbachia* strain infections (Table 4). However, some of this strain diversity within fig hosts is due to the presence of singleton strain infections (although *F*. *arfakensis* subsp.3 additionally contains three *wsp* clade 2 individuals). Ignoring these, specific *Wolbachia* strains are almost totally dominant within individual wasp clades. Predominant *Wolbachia* strains are always below 100% fixation, as uninfected *Wolbachia* associations are often at conspicuous frequencies. Notably, there are repeated examples of dominant alternative strain types (including non‐infections) occupying separate clades within fig host species/complex (i.e., community). Invariably, alternate infection statuses within communities correlate to lowland versus highland population elevations, despite the fact that these wasp species co‐occur when hosts are adjacent.

**TABLE 4:** *Wolbachia* strain associations within fig host species and complexes

Host species complex	Fig host species	Host clade/subspecies	None	C1	C2	C3	C5	C6	C7
*arfakensis*	*F. arfakensis*	subsp.1	5	–	6	–	–	–	–
–	–	subsp.2	9	–	–	–	–	–	–
–	–	subsp.3	10	–	3	15	–	–	–
–	–	subsp.4	15	–	–	1	–	–	–
*itoana*	*F. umbrae*	–	31	–	–	–	1	–	–
–	*F. itoana*	–	4	–	16	1	1	–	–
–	*F. microdictya*	–	7	–	–	–	–	–	28
*trichocerasa*	*F. trichocerasa*	*trichocerasa*	12	–	–	–	1	20	1
–	–	*pleioclada*	1	–	1	–	11	1	‐
*wassa*	*F. wassa*	subsp.1	5	11	–	–	–	–	–
–	–	subsp.2	36	–	–	–	–	–	–

Note

“None” indicates uninfected *Wolbachia* strain associations, while positive strain types are labeled C1, C2, C3, C5, C6, and C7. Ordinal subspecies categories derive from our current assessment (*cf*. *F*. *trichocerasa* has two recognized subspecies).

### 3.2 Simulation of Wolbachia distributions under the “contact contingency” and “adaptive decay” hypotheses

**FIGURE 4:**
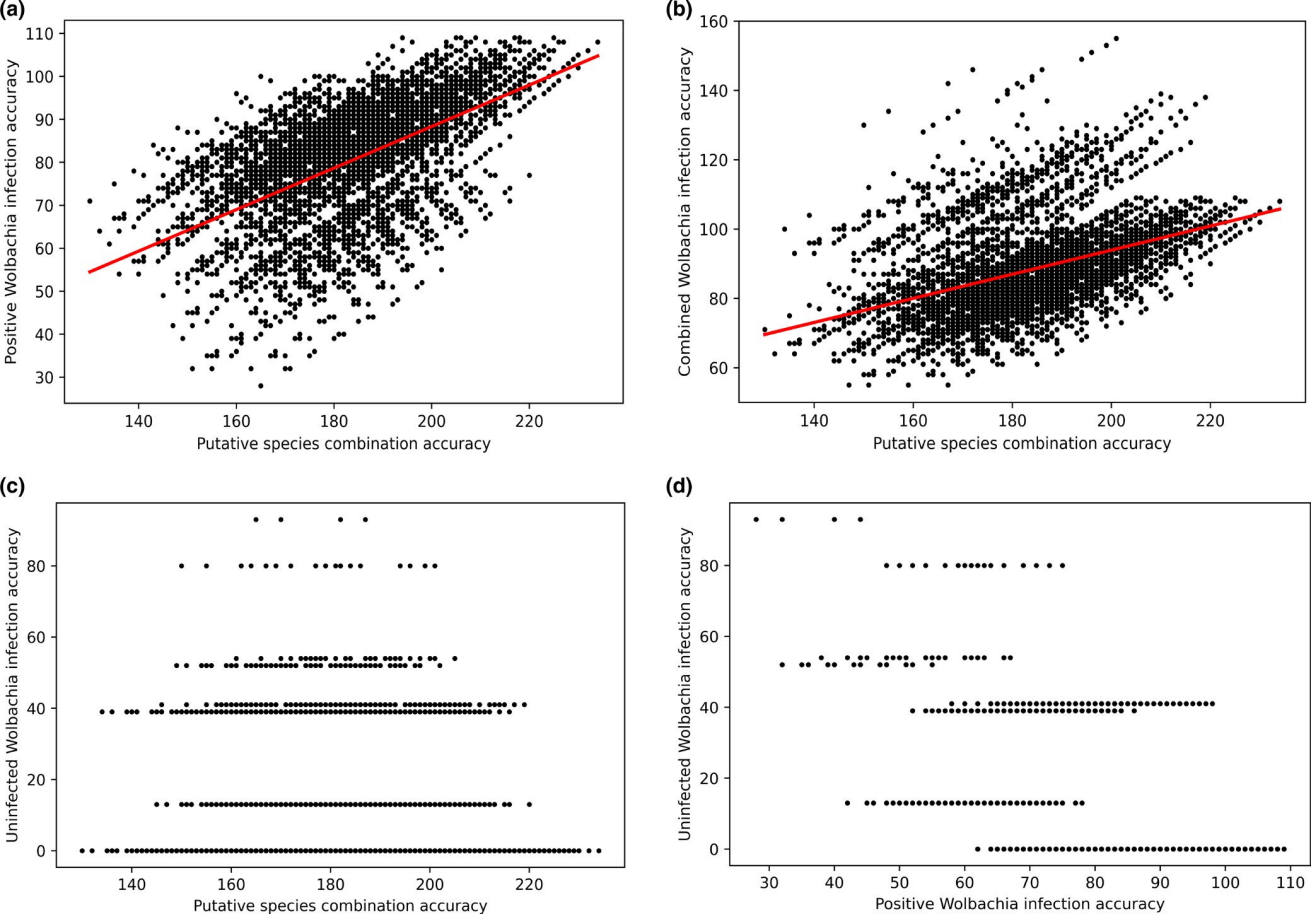
Scatter plots assessing wolpredictor prediction accuracy and the “contact contingency” hypothesis. Individual plots are (a) putative species combination (PSC) accuracy versus positive *Wolbachia* strain accuracy (NB plot shows non‐transformed data), (b) PSC accuracy versus all (positive and uninfected) *Wolbachia* strain accuracy, (c) PSC accuracy versus uninfected *Wolbachia* associations, and (d) positive *Wolbachia* strain accuracy versus uninfected *Wolbachia* associations. PSC accuracy (for each species cluster permutation) calculated against our phylogenetic assessment of species diversity

Evaluating against empirically observed infection statuses (Figure S4), our wolpredictor simulation predicted positive *Wolbachia* infection accuracy (WIA) at up to 91.60% accuracy (109/119 individuals) among putative species combinations (PSCs) featuring 9–11 species (examined range = 6–19 species). wolpredictor also attained high accuracy against empirically derived species richness (89.92%; 107/119 individuals). Regression analyses indicate a highly significant relationship between PSC accuracy (*cf*. empirical species richness) versus wia (*F*_1,8190_ = 5615, adjusted *R* ^2^ = 0.4067, *p* < 2.2e−16; Figure 4a), whilst the relationship weakens when positive (WIA) and uninfected (uWIA) predictions are combined (*F*_1,8190_ = 1933, adjusted *R* ^2^ = 0.1909, *p* < 2.2e−16; Figure 4b). There is no indication of relationships between PSC accuracy and uWIA (Figure 4c), or between WIA and uWIA (Figure 4d).

**FIGURE 5:**
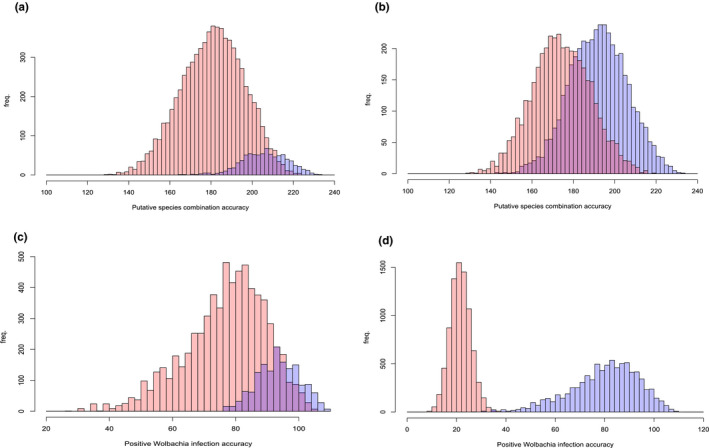
Histograms showing relationships between PSC accuracy and wolpredictor prediction accuracy. Individual plots show: (a) histogram of wolpredictor prediction accuracy associated with the upper 10^th^ percentile of PSC accuracy versus rest of data, (b) histogram of wolpredictor prediction accuracy associated with the 50th percentile of PSC accuracy versus rest of data, (c) comparative histogram of wolpredictor prediction accuracy for species clustering permutations containing the following species richness criteria: *F*. *itoana* (1), *F*. *umbrae* (1), *F*. *microdictya* (1), *F*. *wassa* (≥2), *F*. *trichocerasa* (≥2), and *F*. *arfakensis* (≥2 species), versus the rest of the data, and (d) comparative histogram of wolpredictor positive strain prediction accuracy versus equivalent dataset featuring randomized species clustering

Figure 5a,b indicates the 10th and 50th percentile relationships between PSC accuracy and wia. High‐scoring PSCs show high congruence with features of empirical species richness with highest WIA mostly predicted when species richness counts are: *F*. *itoana* (1), *F*. *umbrae* (1), *F*. *microdictya* (1), *F*. *wassa* (≥2), *F*. *trichocerasa* (≥2), and *F*. *arfakensis* (≥2 species) (Figure 5c). WIA featuring these species cluster iterations have mean 94.10 (79.1%), and a lower 95% confidence interval of 81.10 (i.e., >68.15% accuracy). A two‐sample *t*‐test of all WIA scores (*µ* = 80.53) against results from wolpredictor featuring randomly shuffled wasp clade associations (*µ* = 26.94) shows wolpredictor performs significantly better than chance (*t* = 343.9, df = 9613.6, *p *< 2.2e−16; Figure 5d) on empirical data.

**FIGURE 6:**
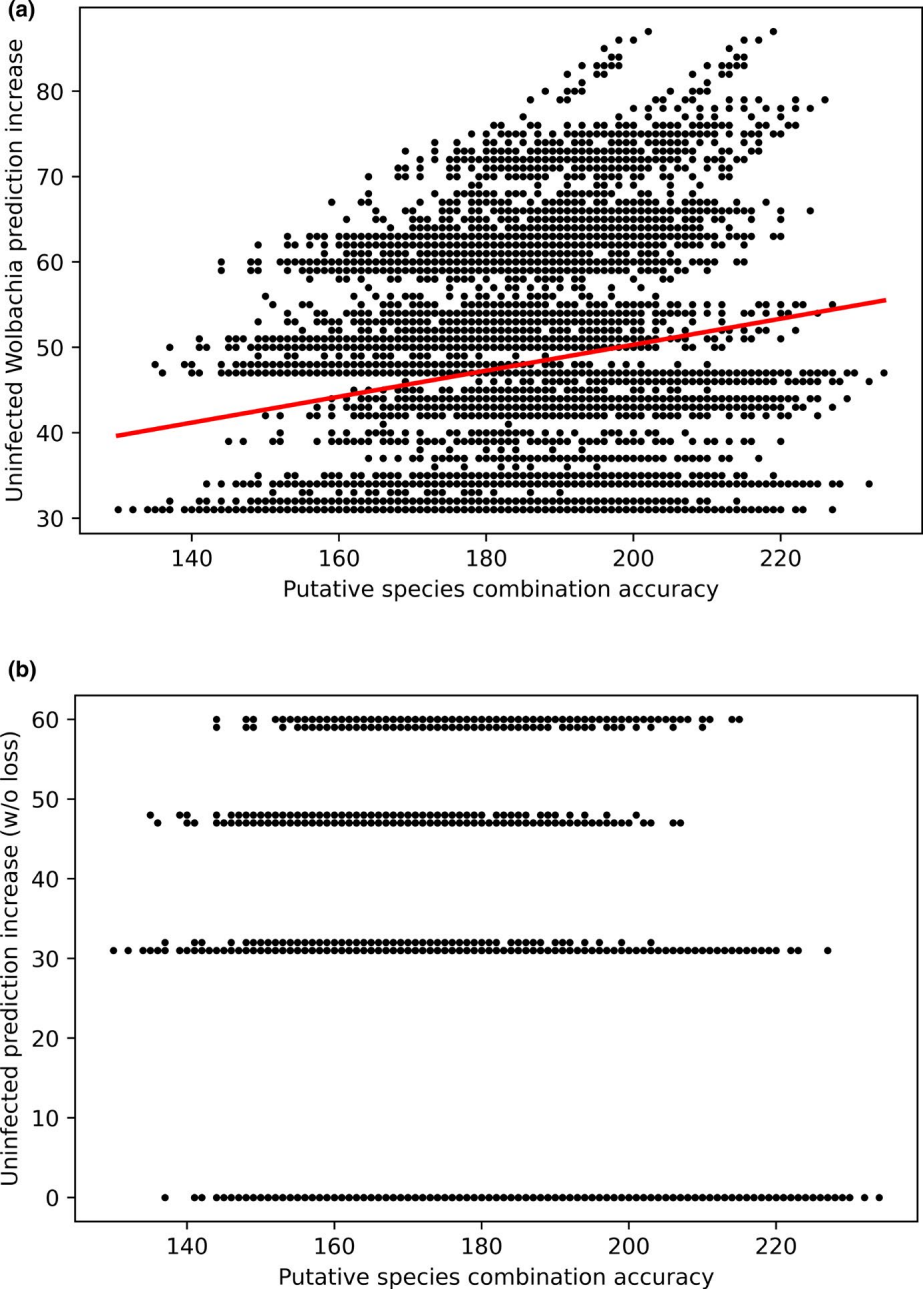
Scatter plots of assessing wolpredictor prediction accuracy and the “adaptive decay” hypothesis. Individual plots are (a) PSC accuracy versus improvements in predicting uninfected associations by purging, (b) PSC accuracy versus improvements in predicting uninfected associations by purging without compromising positive strain association prediction

Addressing our “adaptive decay” hypothesis, wolpredictor improved uWIA up to a maximum of 87 (of 134) individuals, while improvements up to 60 are recorded that do not compromise positive WIA. However, regressing maximum predictive improvement (at each PSC) against PSC accuracy shows weak albeit significant correlation (*F*_1,8190_ = 297.7, adjusted *R* ^2^ = 0.03495, *p* < 2.2e−16; Figure 6a), while there is no indication of a relationship between improvement at each PSC without compromising positive WIA (Figure 6b).

### 3.3 “Fecundity trade‐off” hypothesis evaluation

**FIGURE 7:**
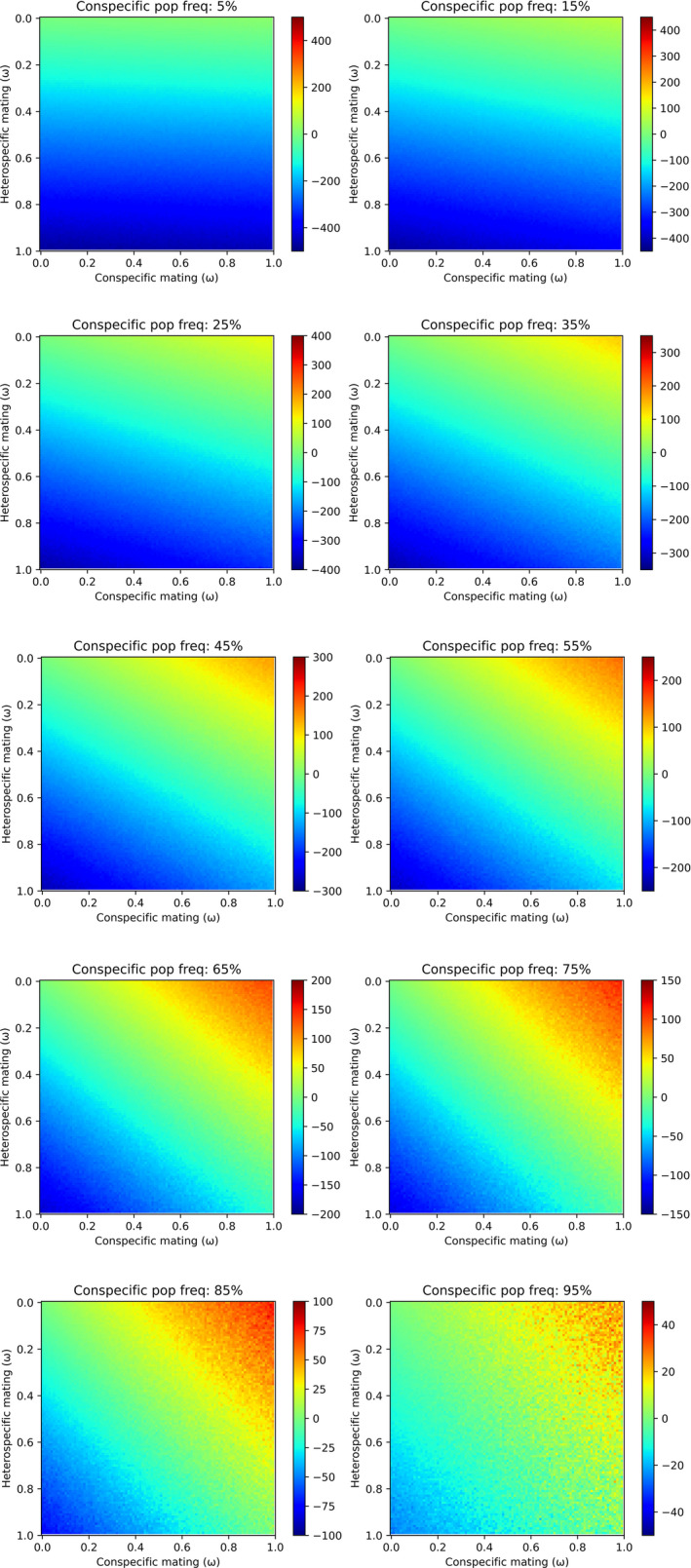
Heat map evaluation of the “fecundity trade‐off” hypothesis. Comparative inclusive fitness values of fig wasp foundresses across relative conspecific‐heterospecific fitness space at different population‐level frequencies of conspecific mating (between 5%–95%) under alternate scenarios of CI‐induced egg mortality (i.e., “*CI*” vs. “*no CI*”). Redder tones (i.e., above zero) indicate relative conspecific‐heterospecific fitness, where foundress inclusive fitness is higher under CI‐induced mortality due to preferential oviposition of higher fitness conspecific offspring despite trade‐offs with fecundity reduction. NB in order to explore all relative fitness space, heatmaps indicate regions where heterospecific fitness is greater than conspecific fitness, which will generally be an unrealistic scenario

Inclusive fitness of individual wasp foundresses differed according to the imposition of CI‐induced egg mortality (Figure 7). As the population level of conspecific mating increases, relative conspecific‐heterospecific mating fitness values begin to favor the CI‐induced egg mortality model (Table 5). Even marginal relative fitness differences between conspecific and heterospecific offspring (e.g., 0.55 vs. 0.45, respectively) result in higher inclusive fitness for foundresses.

**TABLE 5:** Table indicating percentage of pixels where *CI* is favored over *non*‐*CI* according to level of conspecific mating

% conspecifics	CI favored (%)
5	2.18
10	5.16
15	8.46
20	12.12
25	14.63
30	17.52
35	20.89
40	24.78
45	27.05
50	29.78
55	33.08
60	37.32
65	39.2
70	41.47
75	44.88
80	49.85
85	49.85
90	49.94
95	49.34

### 3.4 Evidence for cytoplasmic incompatibility

After filtering, we identified 36,813 hits (matching range: 79%–100%; 540 unique; 90.0% from *cifB*) across *cifA* and *ciB* present in 46 and 97 individuals, respectively (Fig. S5; Table S3). We found *cif* positive/negative to be largely congruent with *wsp*/MLST presence/absence. However, a notable number of wasps showing uninfected *wsp*/MLST associations are *cif* positive (*n* = 36; Table S4). When combined, these push high incidence *wsp*/MLST clades closer to fixation levels, while few *cif* reads are recovered in low incidence *wsp*/MLST clades. Most filtered reads (90.85%) translate without stop codons. For the pollinator of *F*. *arfakensis* subsp. 1, we covered 2720 of 3411 bp of the *cifB* gene across samples (when manually mapping reads) which translates without stop codons, frameshifts, or indels. Assessing coverage based on start‐end positions (*Ceratosolen* hits only) results in 3001 bp coverage out of 3411 bp across all samples.

## 4 DISCUSSION

Understanding the eco‐evolutionary processes regulating the structure of biodiversity is a primary objective in ecology and evolution. Therefore, the current view that arthropod diversity regularly harbors (Weinert et al., 2015) a non‐systematically distributed agent of speciation constitutes a major academic challenge in biodiversity studies. Here, we introduce the “contact contingency” predictive framework for *Wolbachia* strain distributions based on phylogenetic relationships, ecological contact, and host adaptive responses, that shows remarkable accuracy on empirical data. We further examine the impacts of host adaptive primacy on the long‐term evolution of reproductive isolation (RI) via the “adaptive decay” hypothesis that shows moderate ability to predict the decline of *Wolbachia* infections. Finally, our “fecundity trade‐off” model compensates for post‐zygotic fitness reduction imposed by *Wolbachia*—dynamics which would invalidate our proposals without a counter mechanism. We hope our work stimulates further debate around these phenomena. Our approach is particularly suited to the unusual ecology of fig wasps, but they may also operate to differing extents among other ecological systems.

Inspection of our fig wasp phylogeny suggests systemic processes operate because divergent lineages within communities (fig species or complex) at different elevations consistently host nonidentical (including uninfected) *Wolbachia* strains. There are also distinct differences between infection frequencies within communities and some suggestion of horizontal transmission (odd infections on some tips). Analyses of *cifA*/*B* strongly suggest that contrasting infections represent cytoplasmic incompatibility (CI) markers. Around 98.22% of all reads appear functional although the remainder feature stop‐codons. This may be inevitable in large bacterial populations with rapid generation times and does not necessarily indicate non‐functionality. First, the early stop‐codon in some copies of *cifB* may merely slow translation (Shropshire et al., 2021; Wangen & Green, 2020). Second, *cif* genes occur in multiple copies (Martinez et al., 2021) such that some may lose function without loss of CI. Accordingly, alternative functional orthologs were identified for most of the identified stop‐codon reads. Furthermore, Lindsey et al. (2018) showed rapid degeneration of *cif* markers when CI is obsolete. Thus, we would expect rapid and widespread loss of function in organisms without CI, yet we recover almost full‐length functional *cifB* reads (the first gene expected to degrade; Martinez et al., 2021). Finally, multiple mtDNA copies are known from *Ceratosolen armipes* (J.‐Y. Rasplus, personal communication) and other fig wasps (Cruaud et al., 2017) suggestive of *Wolbachia* sweeps. Moreover, our sequencing protocols are suboptimal for insect endosymbionts meaning inference regarding precise functioning cannot be made.

Our data repudiate co‐divergence dynamics, based on vertical transmission, and predominance of horizontal exchange, as individual *Wolbachia* strains invariably do not infect multiple wasp clades occupying the same host species/complex, despite potential for ecological contact. Given associations between an abiotic factor (altitude) and paraphyletic associations between sister host lineages, our data do not obviously support that *Wolbachia* dictates its own infection status (Werren, 2011). Thus, a more parsimonious interpretation is that *Wolbachia* only infects certain insect groups under particular host‐adaptive conditions. A technical note is that our consideration of wider *Wolbachia* diversity (via the global MLST database; Jolley et al., 2018) enables appreciation of host‐endosymbiont phylogenetic incongruence not evident without this context (Figures S2 and S3).

Negative host fitness costs would typically generate a conclusion that *Wolbachia* is the chief architect of its own success. However, it is known that *Wolbachia* sometimes offers host fitness benefits that may have ecological contingencies (Correa & Ballard, 2016; Gavotte et al., 2010), and we do not fully understand the nuances, trade‐offs, and ecological contingencies that determine whether it is circumstantially advantageous. We may consider an insect species that exhibits a broad phenotypic range, say, for ovipositor length, that has bimodal optima according to host plant morphological divergence. Any mechanism preventing reproductive events between extreme phenotypes (yielding intermediate morphs) would be favored providing a net fitness gain.

### 4.1 Evaluating the “contact contingency” hypothesis

Our wolpredictor simulation of “contact contingency” explores a scenario where host tolerance of *Wolbachia* is assumed evolutionarily apposite. Accordingly, adaptively diversifying sister‐species within the same community would be at a selective advantage when harboring alternate strains of *Wolbachia* if they facilitate initial stages of speciation by providing low somatic investment RI. Given that *Wolbachia* infection invariably causes fitness costs (Hoffmann et al., 1990; Perrot‐Minnot et al., 2002), we predict that these patterns are not evident among diverging host lineages not in regular ecological contact (as RI is not required). Finally, because alternative mechanisms of RI may take longer to evolve (Bordenstein et al., 2001; Coyne & Orr, 2004), we introduce the “adaptive decay” hypothesis predicting that species adaptively repel *Wolbachia* infection over extended evolutionary timescales (Bailly‐Bechet et al., 2017; Koehncke et al., 2009).

wolpredictor generates impressive results across our fig wasp data (reaching 91.60% *Wolbachia* infection accuracy for positive strains; WIA) when species delimitation (i.e., putative species combination; PSC) approximates the empirical understanding of species richness. In particular, when single species are predicted within “itoana” complex species, and multiple congeners are predicted across *Ficus arfakensis*, *F*. *trichocerasa*, and *F*. *wassa*, wolpredictor ascribes multiple strains within these communities that reflect the empirical data. Accuracy when considering uninfected *Wolbachia* associations is less precise. This is primarily because uninfected individuals commonly appear among lineages comprising multiple/incipient species within a single host fig (e.g., *F*. *wassa* ‐ infection rate = 21.15%), where wolpredictor ascribes positive *Wolbachia* associations. Potentially, non‐infection in these lineages may result from evolved redundancy (adaptive decay) of CI.

Our framework, like others, is imperfect and demands rigorous testing using additional data sets where parameters may be more freely varied. Our approach is designed to test theoretical expectations in a predictive manner using empirical data. Although we accept that more extensive formal modeling and parameter simulation may also provide more insight considering that many parameters are derived from the data.

### 4.2 Evaluating the “adaptive decay” hypothesis

To consider the “adaptive decay” hypothesis, wolpredictor removes *Wolbachia* from lineages where pairwise branch length distances exceed thresholds representing evolutionary time. It may constitute a crude method when uniformly applied across lineages. Whilst occasionally yielding marked improvements in predictive ability for uninfected samples, it does not improve performance systematically to suggest successful modeling of biological processes. This maybe because alternative RI mechanisms (rendering *Wolbachia* redundant) may appear at different rates across lineages due to functional genomic variation or unconsidered ecological contingencies—in fig wasps, syconia access is partially controlled by relative syconia‐wasp size (Bronstein, 1987), which mechanically prevents hybridization among some species.

Furthermore, under a simple expectation of panmixis and infinite population size, CI is predicted to sweep to fixation, contrary to the population level polymorphism in our data. However, this depends on perfect transmission, and infection rates may decay even if fixation is achieved (Engelstädter & Telschow, 2009). Moreover, hymenopteran haplodiploidy can facilitate the survival of infected haploid males (Breeuwer & Werren, 1990), which alongside inbreeding can result in higher invasion thresholds and reduced stable equilibrium frequencies (Engelstädter & Hurst, 2006b). These considerations deserve further attention, but may explain observed infection frequencies below fixation alongside additional *cif* sequencing levels that suggest augmented/hidden levels of CI among high incidence *Wolbachia* clades (likely due to differential sequencing platform sensitivities; see Table S4 and Wolfe et al., 2021). The reduced level of *cif* read recovery among low incidence clades adds to the impression that decay may be ongoing in some clades.

Overall, our rules‐based wolpredictor algorithm captures much embedded structure from a dataset presenting a superficially stochastic appearance. Thus suggesting that environmentally contingent symbiotic benefits (Correa & Ballard, 2016) systematically sum to yield predictable *Wolbachia* distributions. However, we should consider the impact of community delineation regarding the “itoana” complex as a single community (following Souto‐Vilarós et al., 2019) that expedites high accuracy (Figure 5c). Alternatively, we may consider three distinct *Ficus* communities wherein wolpredictor would ascribe non‐infection statuses across all wasps when evaluating single wasp species communities. This point emphasizes wolpredictor’s sensitivity to species delimitation (PSC) and community boundaries and intimates that high accuracy seems only likely if *Wolbachia* functionality (*viz*. CI) and other system dynamics mirror our proposals.

### 4.3 Evaluating the “fecundity trade‐off” hypothesis

Our framework may be considered theoretically problematic since CI is a post‐zygotic mechanism causing immediate fitness costs in host fecundity that must be overcome (Caspari & Watson, 1959). However, the unique life histories and ecological conditions of fig wasps means they may tolerate CI: oviposition sites are at especially high premium (Dunn et al., 2015), fig wasps are known to produce surplus eggs (Dunn et al., 2011), and co‐evolved species are renowned for precise tolerances in interacting traits that render hybridization particularly costly (Weiblen, 2004). Indeed, in contrast to their host figs, wasps form well‐defined species (Souto‐Vilarós et al., 2018, 2019). Thus, we investigated the impact of CI when considering the oviposition constraints of fig syconia. We show that inclusive fitness of multiple‐mated CI females can be higher providing reduced egg‐load and/or selective ovipositioning facilitates strategic utilization of higher‐quality fig ovules less vulnerable to parasitoid attack (Dunn et al., 2008). Results support the hypothesis that fig wasps may adaptively evolve CI through traits to harbor *Wolbachia*.

The interaction of CI on multiple‐mated fig wasps has not been studied but mechanisms that could facilitate our oviposition simulation have been identified in *Drosophila* via *Wolbachia* associated reductions in sperm competition abilities (Champion de Crespigny & Wedell, 2006) and egg load reductions (Weeks et al., 2007). Given reproductive manipulations of haplodiploid Hymenoptera such as selective fertilization and sex‐ratio adjustment (discriminated by ploidy), it is entirely plausible that appropriate mechanisms operate in fig wasps. Further, extreme fig wasp sib type competition for developmental resources may occur in other taxa. Thus, our model suggesting post‐zygotic fitness losses do not necessarily sum to net negative fitness may be generalizable beyond Hymenoptera. For fig wasps, future work examining whether CI results in differential pre‐oviposition embryo mortality, and whether selective oviposition of conspecific versus heterospecific eggs occurs, is required.

### 4.4 Implications

Our approach diverges from some conventionally held opinions regarding *Wolbachia* host manipulation (e.g., Werren, 2011) and net conflict with hosts (e.g., Charlat et al., 2007), but often conform with evolutionary expectations of CI systems. Customarily, post‐zygotic fitness losses must be balanced by some rescuing dynamic (Caspari & Watson, 1959; Turelli, 2010; but see Turelli & Hoffmann, 1991). Our “contact contingency” hypothesis assumes CI via host‐adaptive divergence, while our “fecundity trade‐off” framework suggests intense offspring competition critically mitigating the “Jekyll and Hyde” dynamics (*sensu* Jiggins & Hurst, 2011) of post‐zygotic mortality. Ongoing gene flow derives from migration rate and magnitude of differential selection (Telschow et al., 2002). In co‐evolutionary systems, extreme functional trait matching may magnify the effects of divergent selection, favoring our fig‐wasp paradigm. Furthermore, *Wolbachia* infections dissipate over extended timescales (Bailly‐Bechet et al., 2017). Our “adaptive decay” hypothesis postulates alternative RI mechanisms select for host‐adaptive purging (which typically receives equivocal support; Koehncke et al., 2009), that wolpredictor fails to consistently capture in relation to phylogenetic structure. We explicitly incorporate bidirectional‐CI, but unidirectional‐CI may also promote speciation (Telschow et al., 2007). Our ability to predict decay/absence (in largely uninfected clades) may benefit from incorporating unidirectional dynamics into our models, although low‐frequency recovery of *cif* markers among some clades suggest bidirectional‐CI undergoing decay could as parsimoniously explain these patterns—our study represents a snapshot in time after all.

Overall, we contend that it is difficult to propose an alternative systemic framework that describes our, or other published community datasets, or assert that observed structural patterns are stochastically generated. That is, given *Wolbachia* is maternally inherited and that occasional incidences of horizontal transfer suggest its potential pervasiveness, why do we see alternate infection patterns if *Wolbachia* infection abilities trump the interests of hosts? Among malvantheran fig wasps, communities featuring singleton congeners invariably display uninfected *Wolbachia* associations, while the reverse is true where multi‐congeners co‐occur (Haine & Cook, 2005). Additionally, *F*. *benjamina* wasps display “chaotic” *Wolbachia* associations, including among congeners (Yang et al., 2012). However, we would also state that we do not expect all incidences of CI across the arthropod phylogeny to result from these dynamics.

Critically, for most global diversity, we simply do not have the detailed ecological information to reliably evaluate the processes underpinning community assembly (Segar et al., 2020). There is growing consensus that investigations of biodiversity need to consider interactions both within and between all trophic levels whilst also discriminating significant versus trivial dynamics (Segar et al., 2020), or, more generally, ecological contingency, whose agents may be bacterial, fungal or viral in origin. Failure to account for these factors may mean we never fully disentangle the myriad determinants of ecosystem dynamics nor quantify the relative contributions of stochastic (*viz*. neutral; Hubbel, 2001) processes.

## 5 CONCLUSIONS

Our results indicate that *Wolbachia* distributions are systematically structured among an arthropod dataset based on a predictive framework invoking adaptive responses in host fig wasps. A parsimonious interpretation of these findings suggests that ecologically contingent co‐evolutionary benefits of *Wolbachia*‐induced CI, with respect to adaptive lineage diversification, systematically sum to yield predictable distributions despite initial appearances that the endosymbiont is stochastically distributed. Our data suggest that future work assessing biodiversity patterns among arthropods should incorporate *Wolbachia* infection data (alongside other microorganisms) as an added dimension to account for potentially confounding variables. Our aim is to stimulate debate and subsequent research in unravelling a rather puzzling phenomenon within arthropod biodiversity.

## CONFLICT OF INTEREST

The authors declare no conflicts of interest.

## AUTHOR CONTRIBUTIONS

**Clive T. Darwell:** Conceptualization (lead); Formal analysis (equal); Methodology (lead); Software (lead); Writing – original draft (lead); Writing – review & editing (lead). **Daniel Souto‐Vilaros:** Data curation (lead); Formal analysis (lead); Writing – review & editing (supporting). **Jan Michalek:** Data curation (lead); Methodology (lead). **Sotiria Boutsi:** Data curation (supporting); Writing – review & editing (supporting). **Brus Iusa:** Data curation (supporting). **Mentap Sisol:** Data curation (supporting). **Thomas Kuyaiva:** Data curation (supporting). **George Weiblen:** Project administration (supporting); Supervision (supporting); Writing – review & editing (supporting). **Vlastimil Křivan:** Data curation (supporting); Methodology (supporting); Writing – review & editing (supporting). **Vojtech Novotny:** Funding acquisition (lead); Project administration (lead); Resources (lead); Supervision (supporting); Writing – review & editing (supporting). **Simon T. Segar:** Conceptualization (supporting); Data curation (supporting); Formal analysis (equal); Funding acquisition (lead); Methodology (supporting); Project administration (lead); Resources (lead); Supervision (lead); Validation (lead); Writing – original draft (supporting); Writing – review & editing (equal).

### OPEN RESEARCH BADGES

This article has been awarded Open Data, Open Materials Badges. All materials and data are publicly accessible via the Open Science Framework at https://github.com/ctdarwell/wolPredictor; https://www.ncbi.nlm.nih.gov/sra?linkname=bioproject_sra_all&from_uid=555181.

## Supporting information

Supplementary Material

## Data Availability

github repository for wolpredictor computer code and figwasp and *Wolbachia* metadata: https://github.com/ctdarwell/wolPredictor. *Wolbachia* sequence data: Genbank accessions ON095348–ON095736. RAD‐seq accession numbers originally published with Souto‐Vilarós et al. *Mol Ecol*. **28**:3958–3976: MN168894–BMN169018.

## ENDNOTE

^1^
We order the *n* elevation sites according to their elevation in an increasing order. Putative species can be grouped into clusters according to the number of different species in each cluster. The number of ways putative species can be grouped into *k* putative species in the community is $\left(\begin{array}{c} n‐1\\ k‐1 \end{array}\right),\;k=1,\ldots ,n‐1$. For example, if there are *n* = 4 sites, there are $\left(\begin{array}{c} 3\\ 1 \end{array}\right)=3$ combinations of two putative species in the community {{1000,0111},{1100,0011},{1110,0001}}. To calculate the number of all possible combinations of putative species in the community we sum up the numbers of combinations across all *k's*, that is, $\sum_{k=1}^{n‐1}\left(\begin{array}{c} n‐1\\ k‐1 \end{array}\right)=2^{n‐1}$.
