# Supplementary material for: Predicting distributions of Wolbachia strains through host ecological contact—Who's manipulating whom?
Source: Ecol Evol. 2022 Apr 13;12(4):e8826. doi: 10.1002/ece3.8826 (PMC9006231; doi:10.1002/ece3.8826)
Supplement: Supplementary file 1 — Supplementary Material [file ECE3-12-e8826-s001.docx]

Figure S1a)


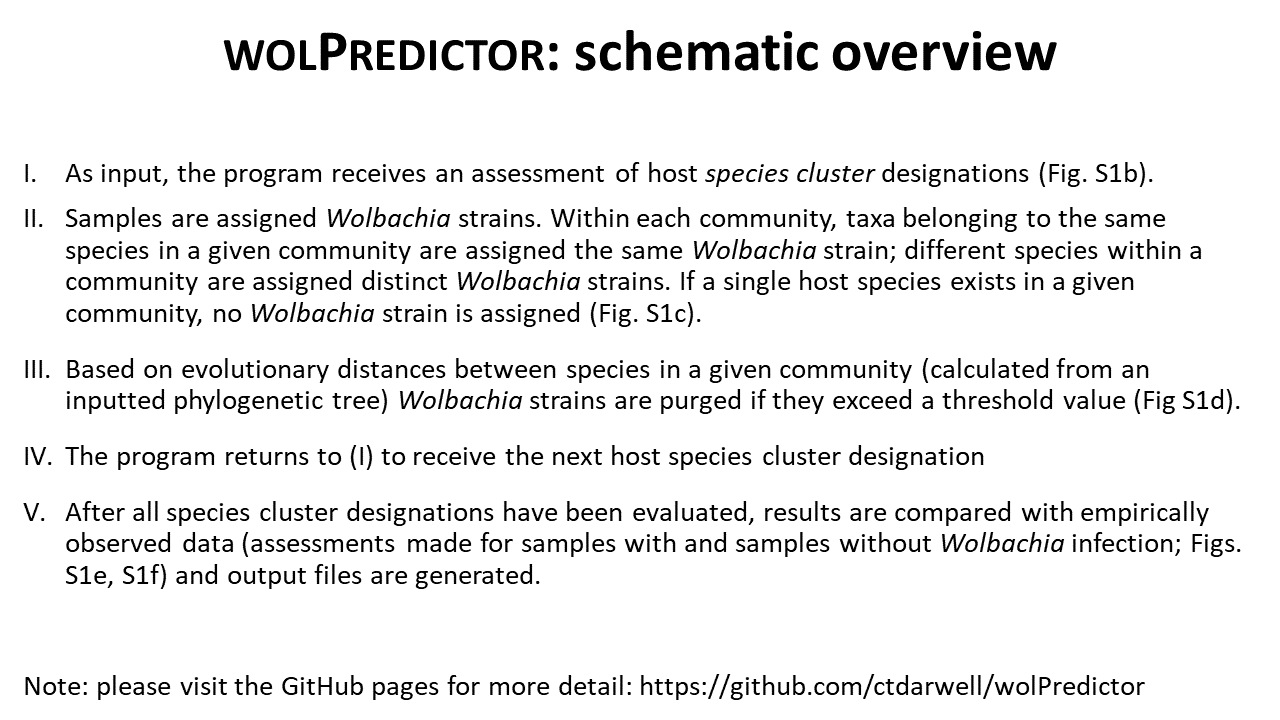


Figure S1b)


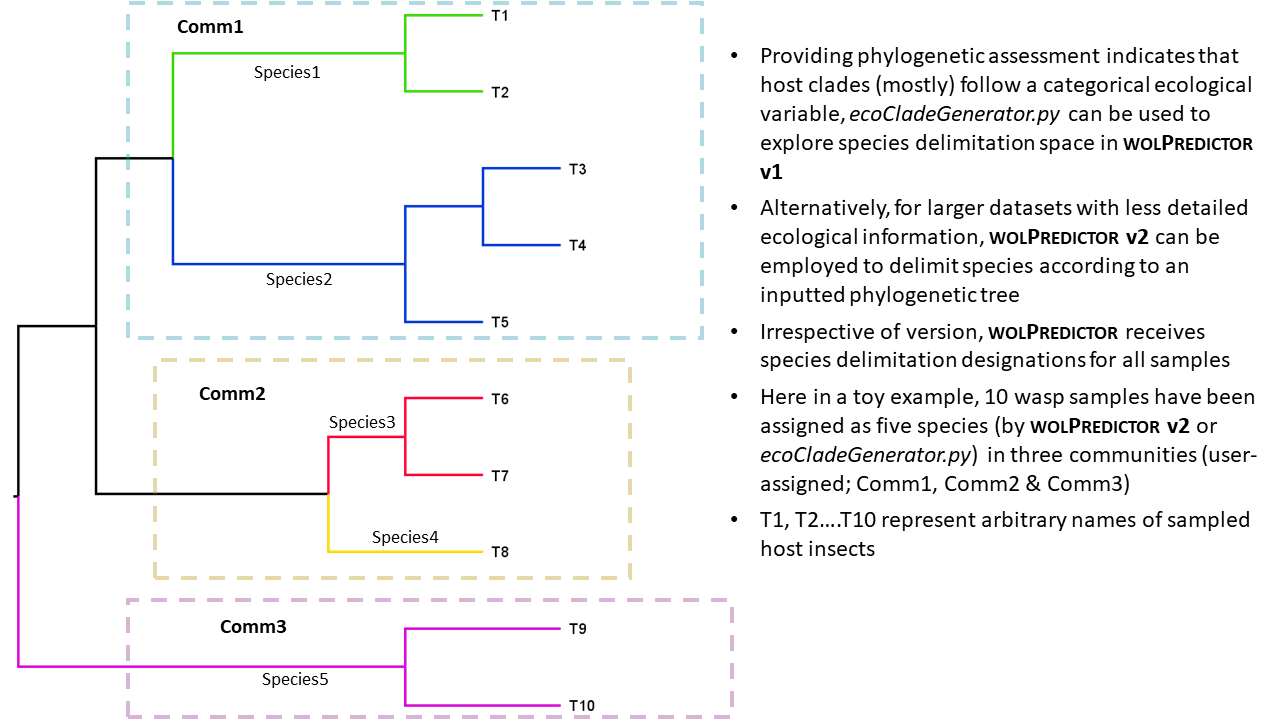


Figure S1c)


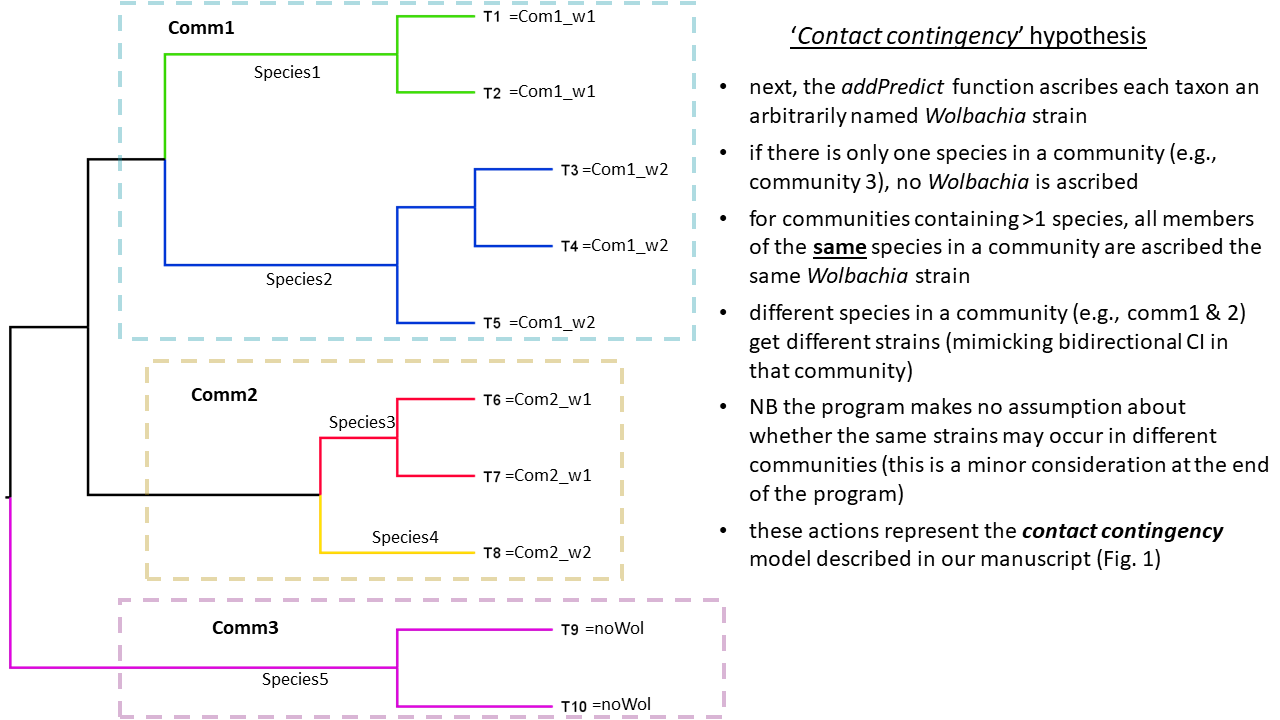


Figure S1d)


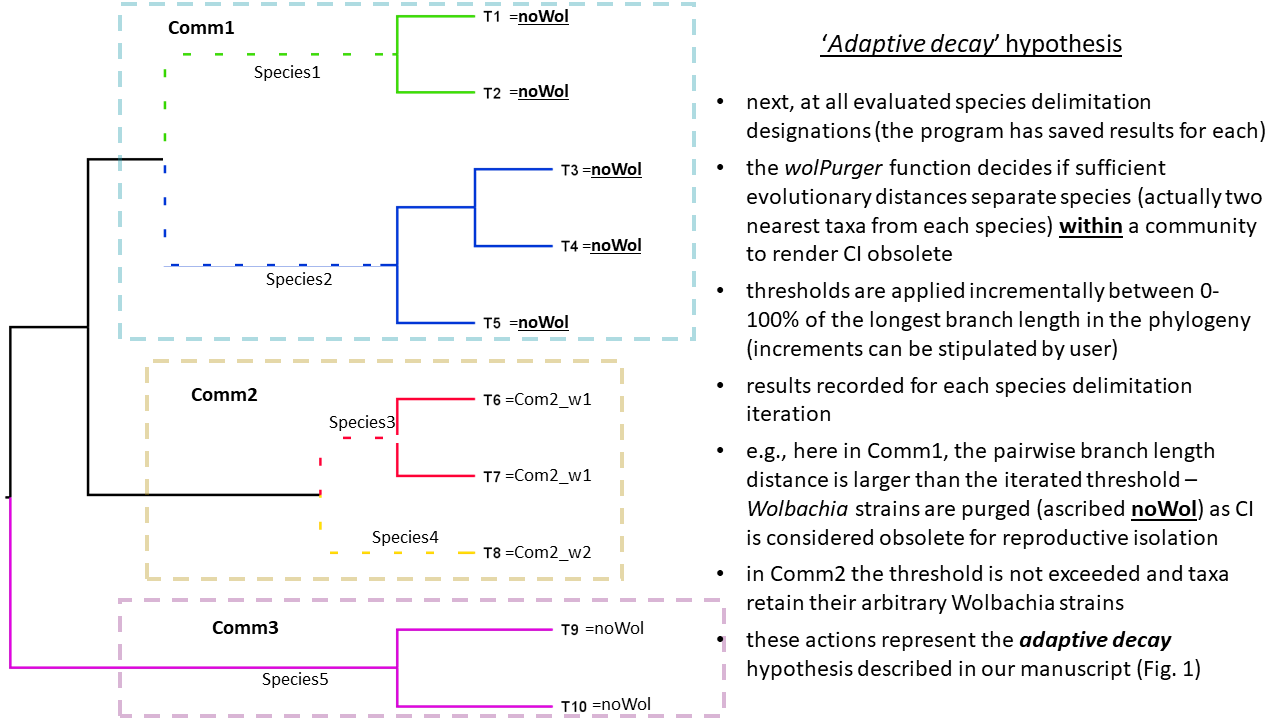


Figure 1e)


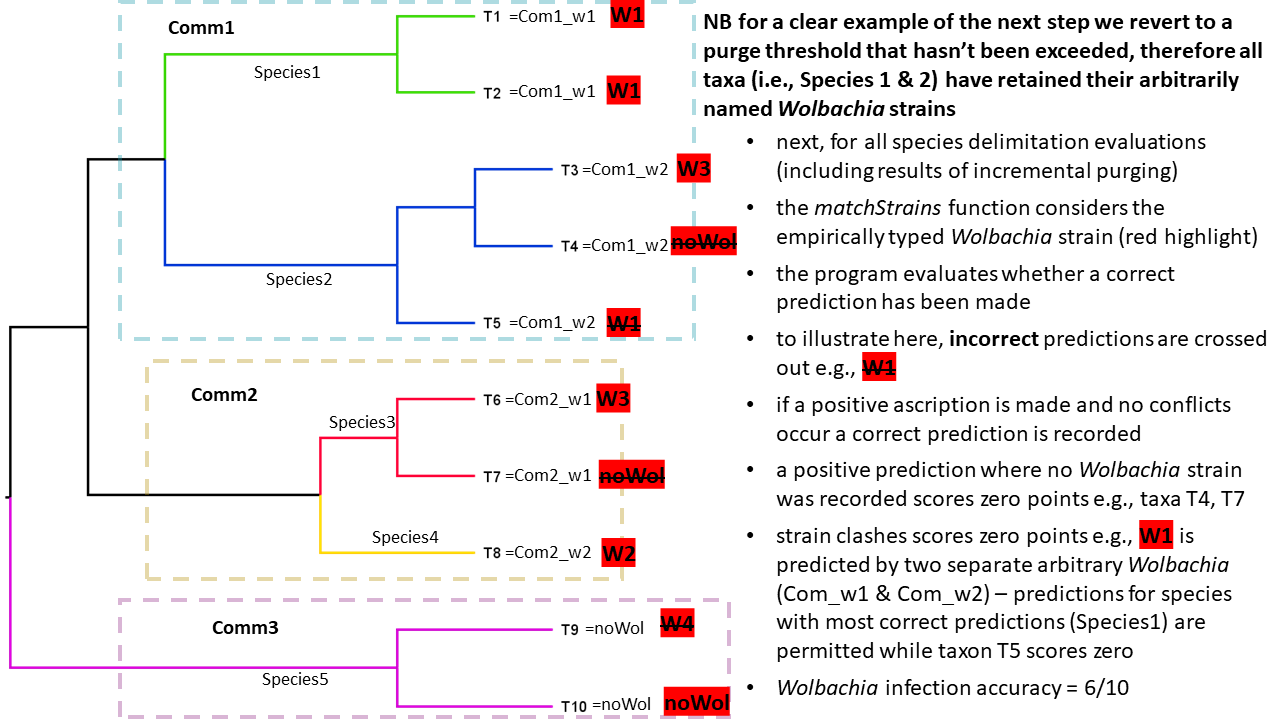


Figure S1f)


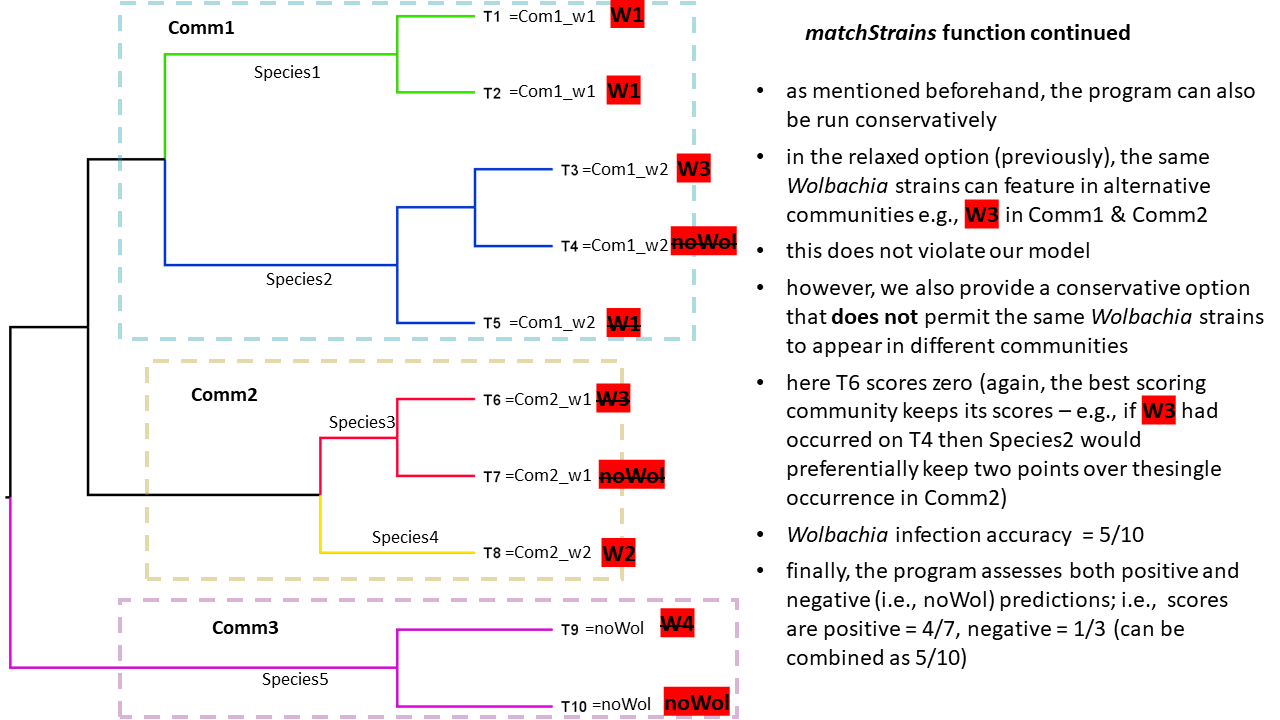


**Figure S1.** Stepwise schematic overview of the wolPredictor algorithm built in Python v.3.6.9. The behaviour of wolPredictor across a toy dataset is examined.

Figure S2a)


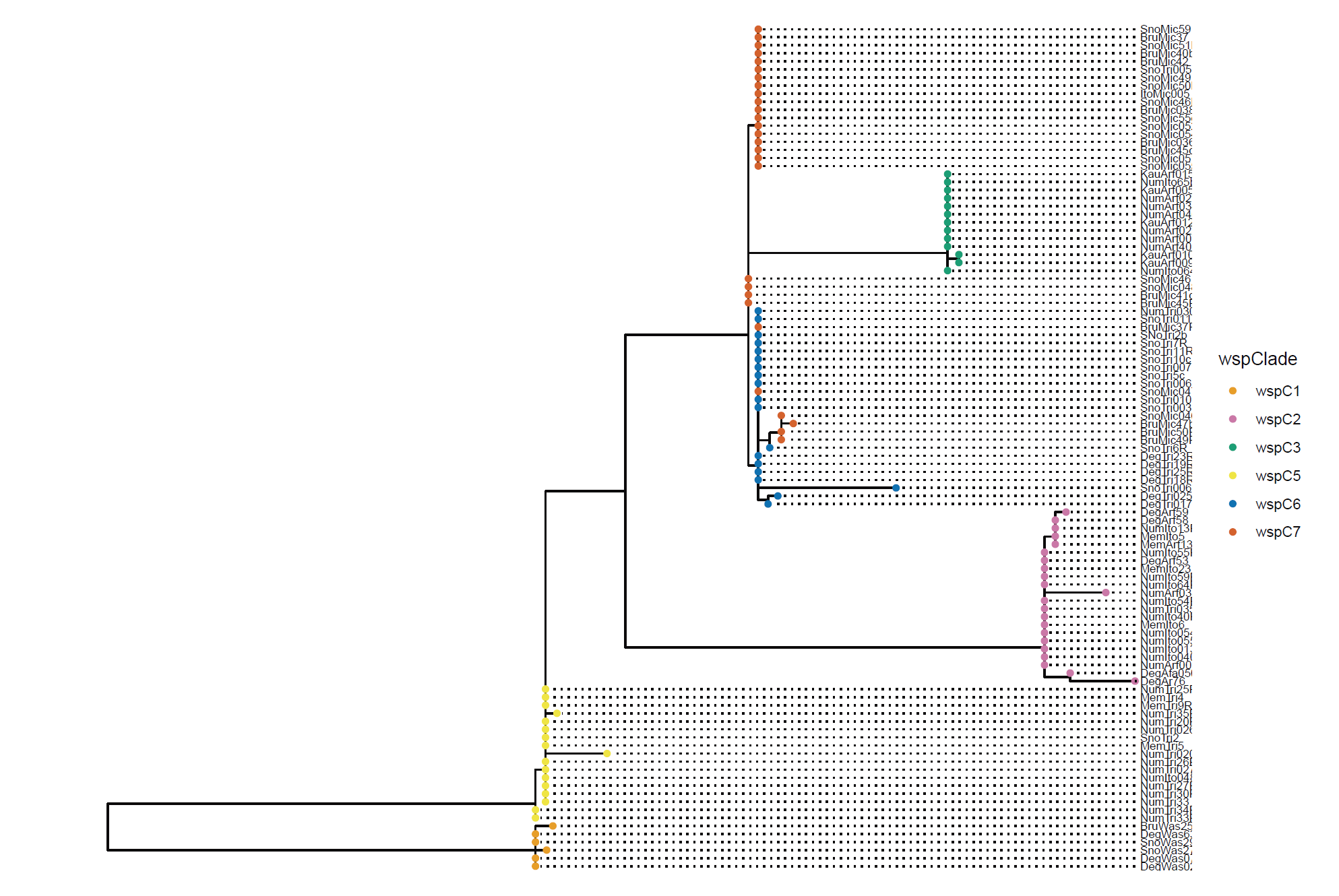


b)


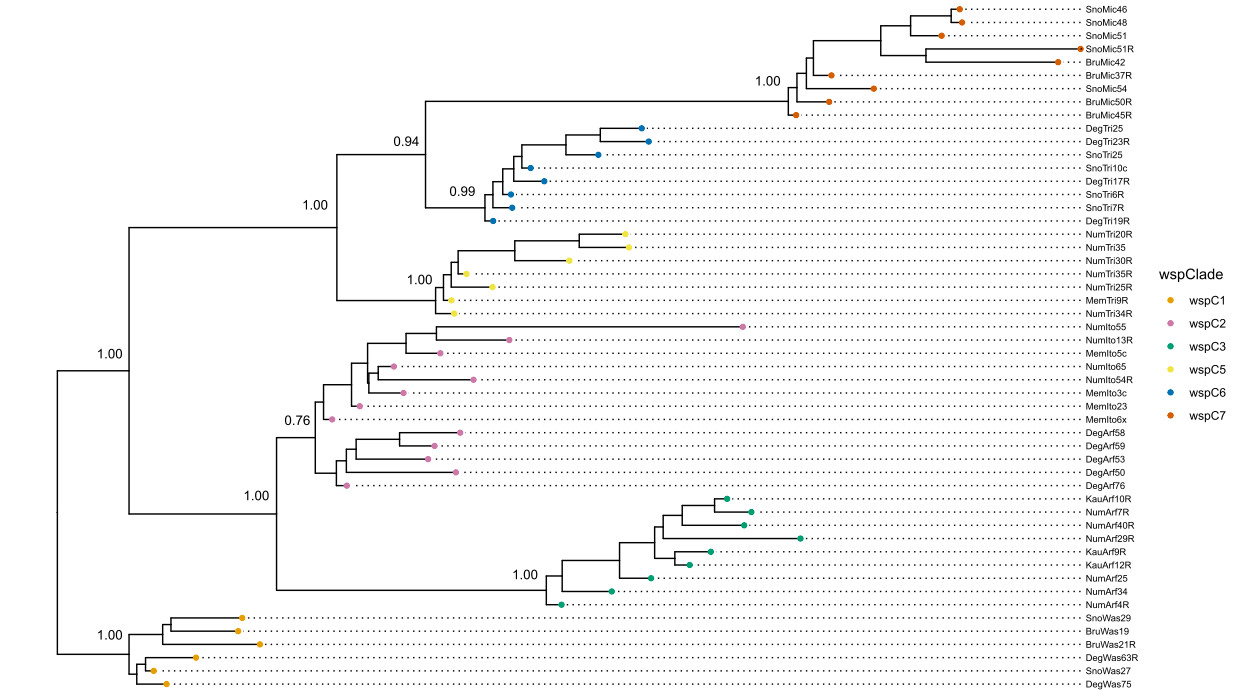


c)
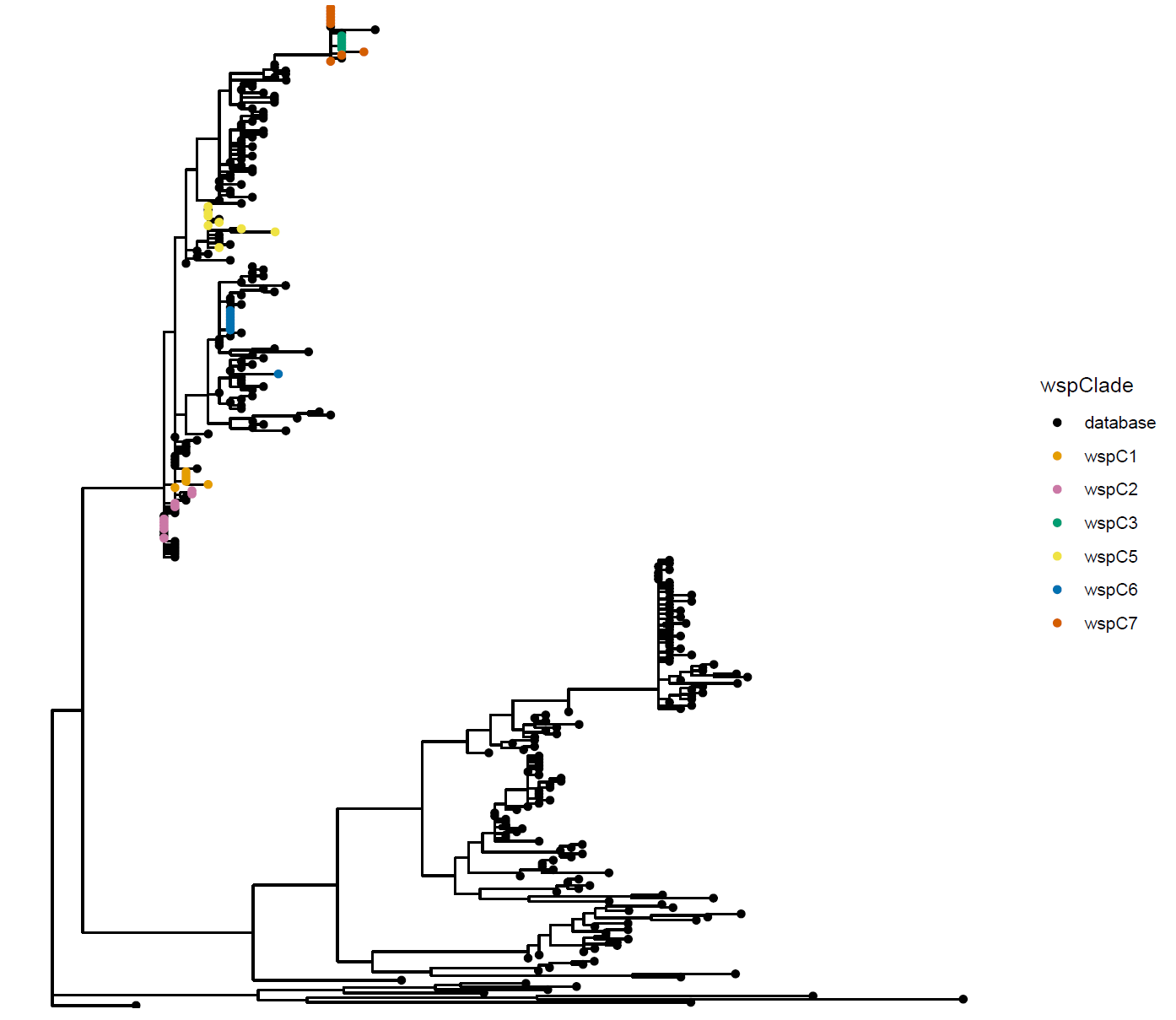


d)


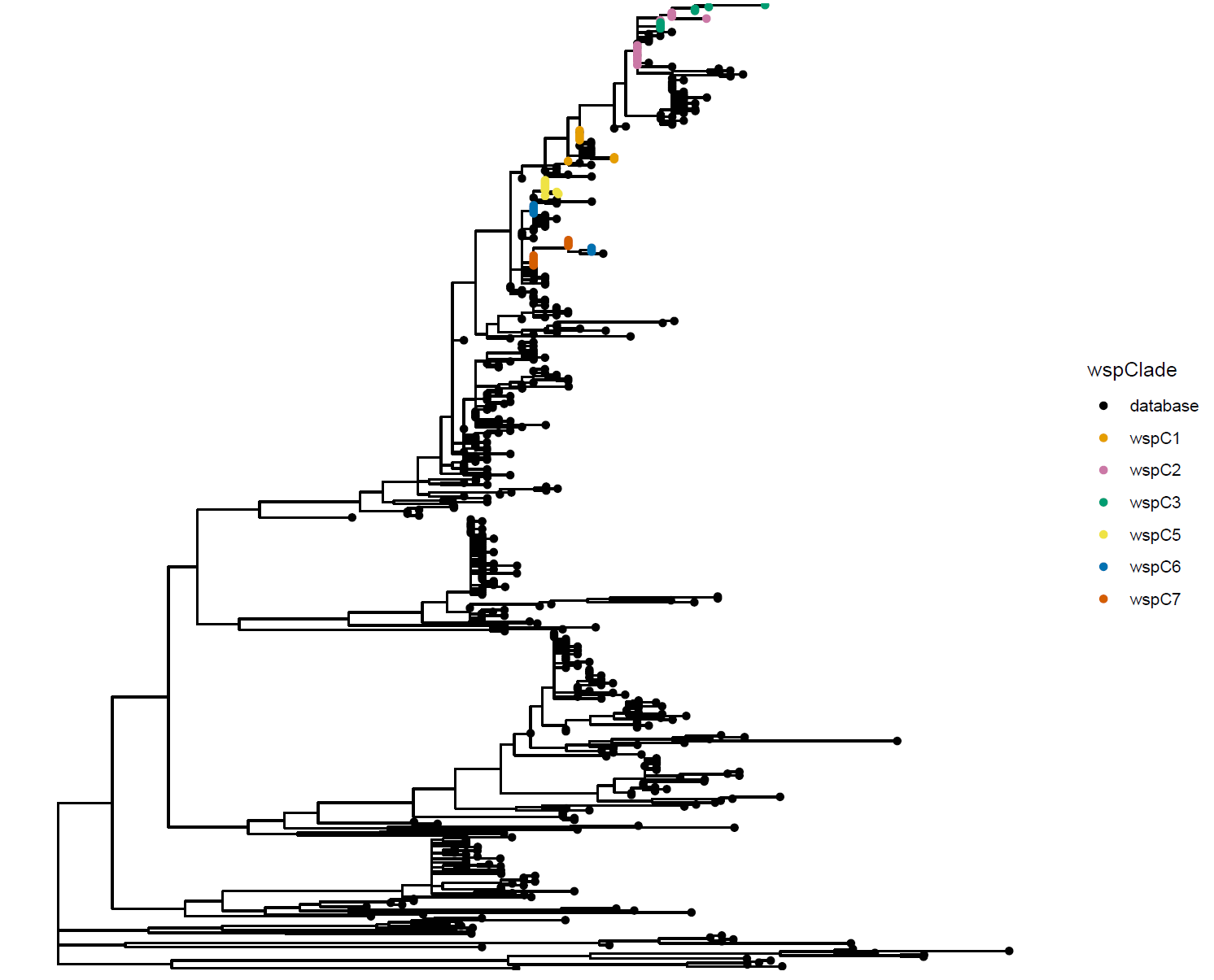


e)


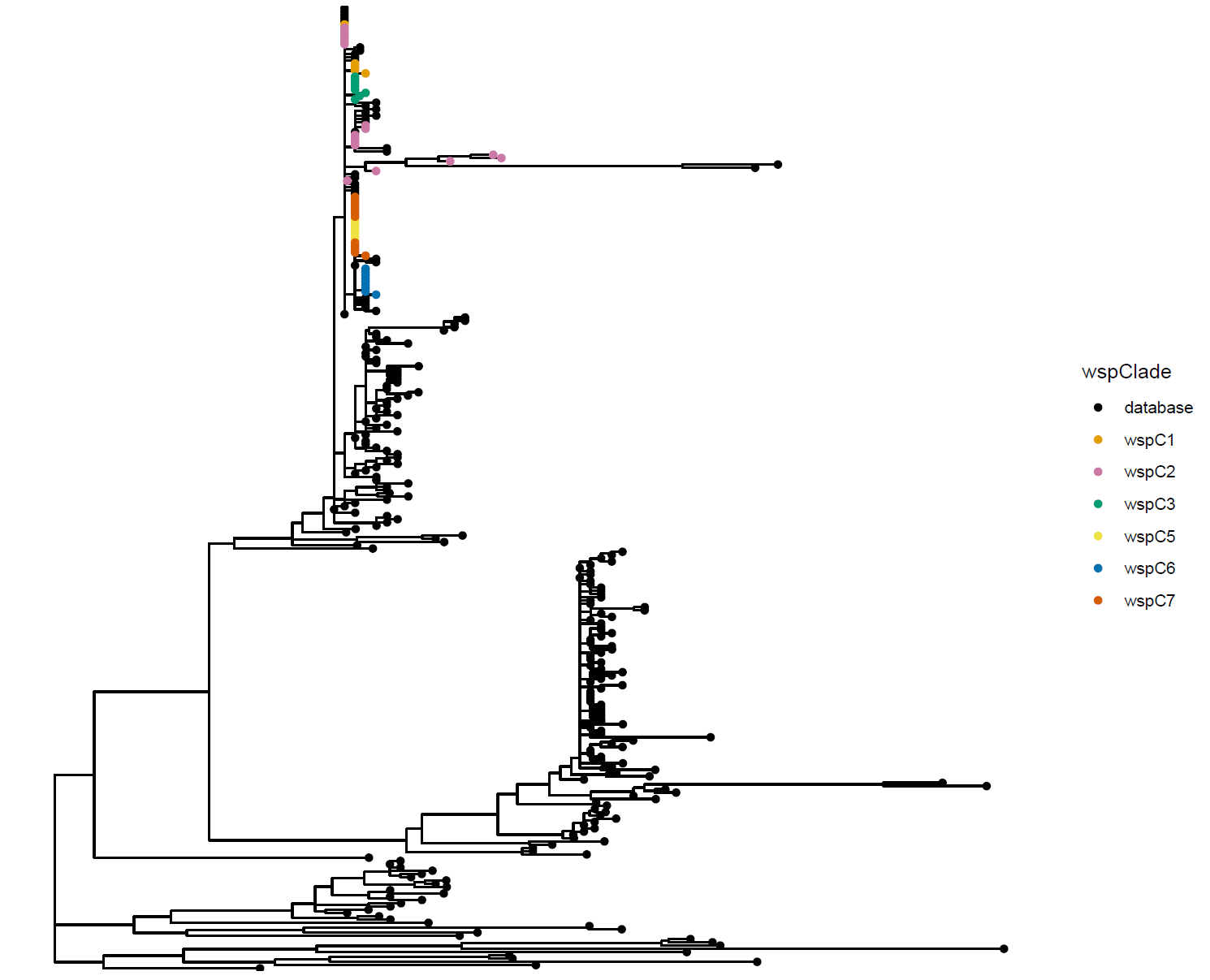


f)


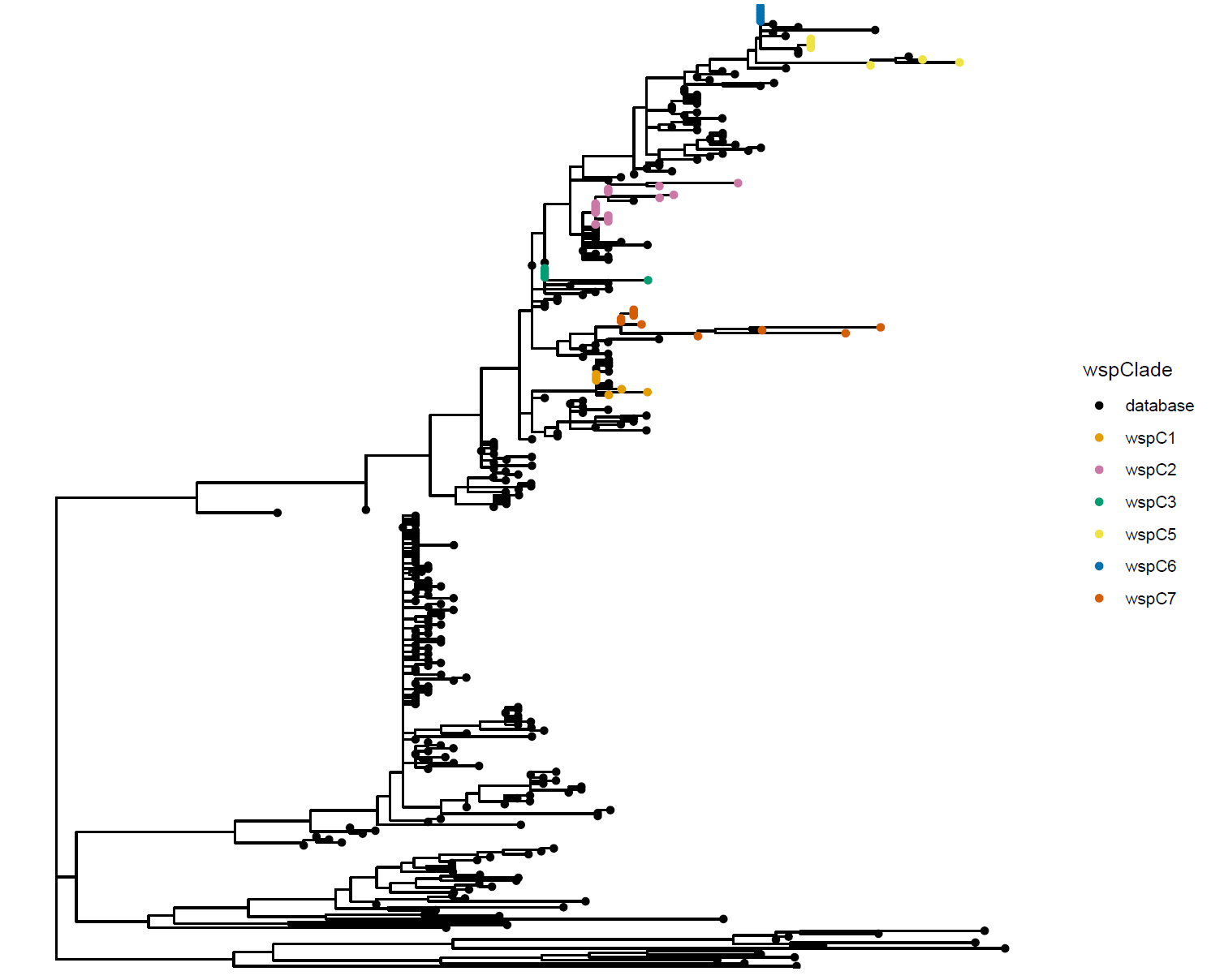


g)


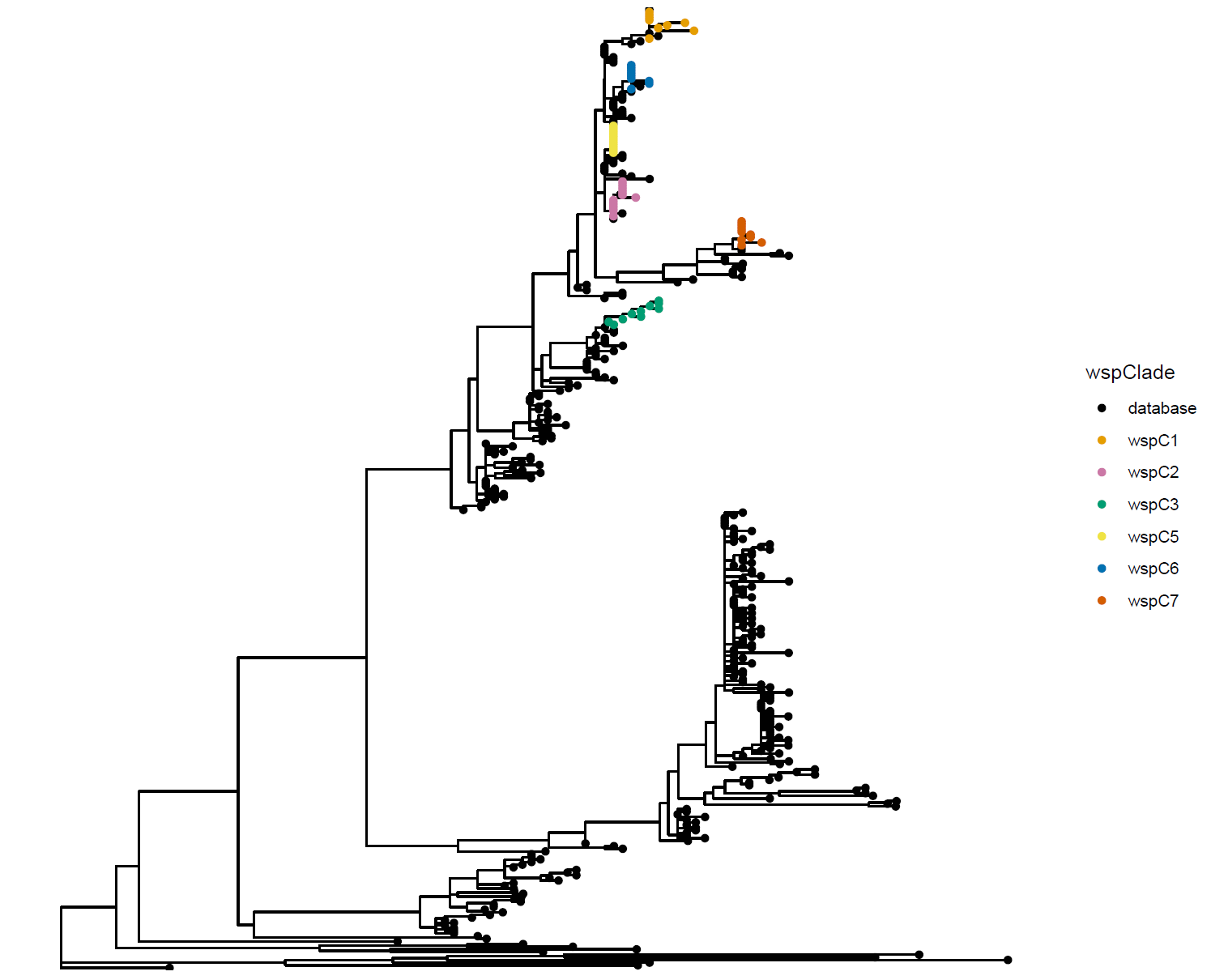


h)


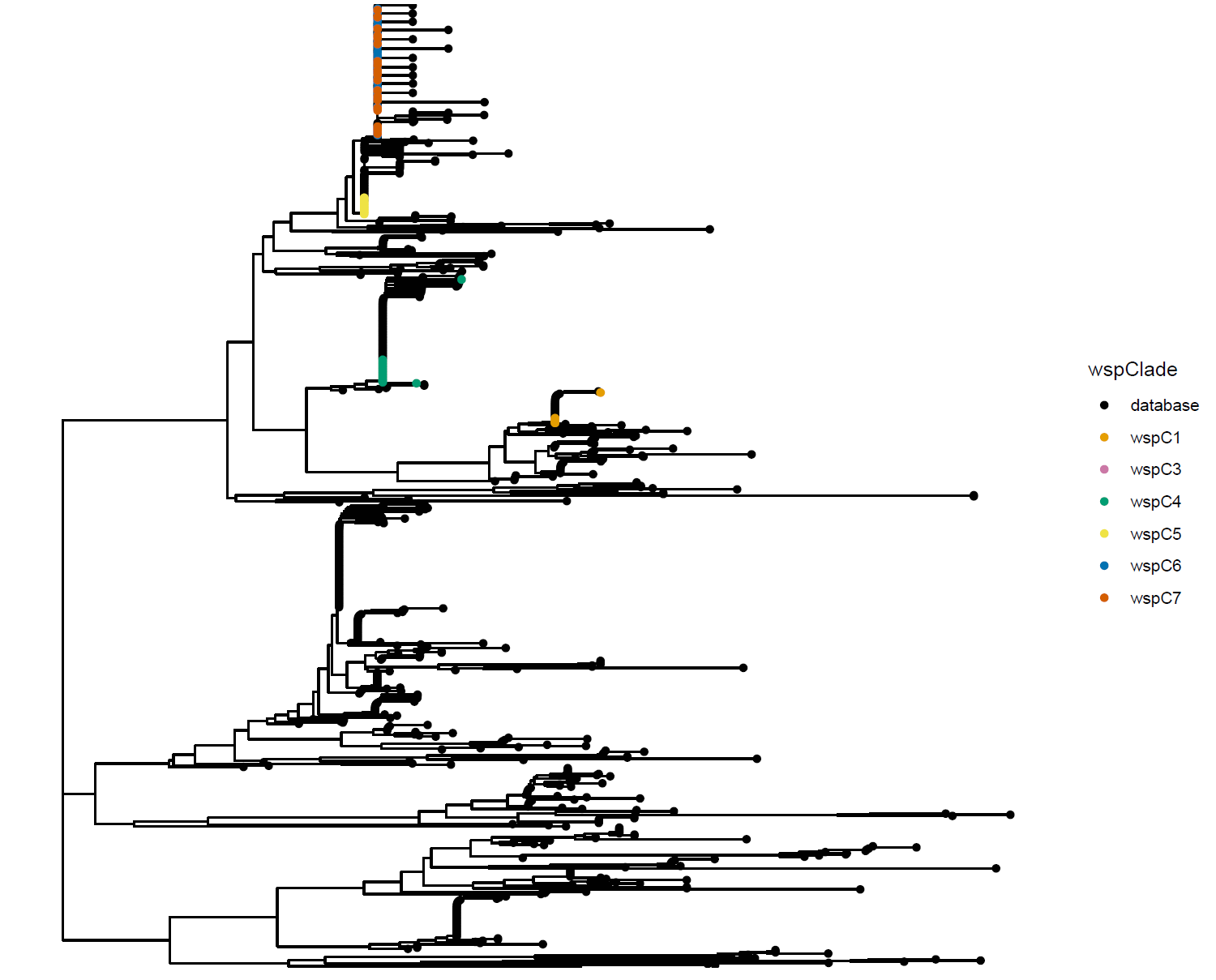


**Figure S2**. A *wsp* single gene phylogeny for sequences generated in this study (a) and (b) a Bayesian phylogeny derived from the five MLST genes for sequences from this study only (both rooted to *Wolbachia* extracted from a species of *Pleistodontes* pollinating fig wasp sampled Mt Wilhelm) and c) Single Maximum Likelihood phylogenies for c) *coxA*, d) *fbpA*, e) *ftsZ*, f) *gatB*, g) *hcpa* and h) *wsp* including sequences generated for this study and all accessions from the MLST data base. Colour coding depicts *Wolbachia* strains orange: wspC1, pink: wspC2, green: wspC3, yellow wspC5, blue: wspC6 and red: wsp7. Note that wsp6 and wsp7 are not distinguishable using *wsp* alone. All of these figures are available as PDFs in the electronic appendix. Where given labels give Bayesian posterior probability support.


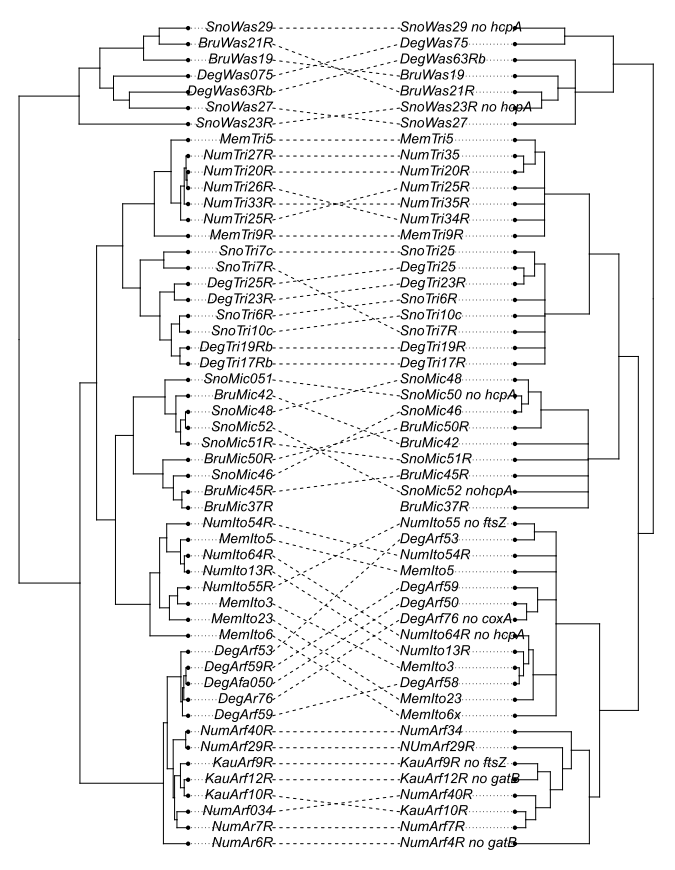


**Figure S3**. A co-phylogeny plot using the Bayesian molecular phylogenies for pollinating fig wasps (left) and MLST (right). Congruence between phylogenies was tested statistically using Procrustes Application to Cophylogenetic Analysis as implemented in the R package ‘paco’ using the ‘r0’ permutation method and 1000 permutations. Congruence was significant (ss= 19.106, p<0.0001). However, these strains do not share a most recent common ancestor in most cases, thus congruence here is unlikely to reflect co-diversification. We generated time-calibrated ultrametric phylogenies through penalised likelihood as implemented in the ‘chronos’ function in the R package ‘ape’ (version 5.5) (Paradis et al., 2021). In preparation we multiplied the edge lengths (in substitutions per site) by the number of bases in the alignment to get values in substitutions. The value of ƛ (the smoothing parameter) was estimated using penalised likelihood and cross validation (using R function ‘chronopl’ with a range of 10e-1 to 10e+6), the value with the lowest cross-validation score was selected (10e-1) (Sanderson, 2002). We calibrated our phylogenies using a minimum root age of 1. Finally, we used the function ‘chronos’ to produce a time-calibrated phylogenies using four models: “relaxed”, “correlated”, “discrete” and “strict” (Kim & Sanderson, 2008; Paradis, 2013), these were compared using penalised likelihood with the best chosen model being the “relaxed” model in both cases.


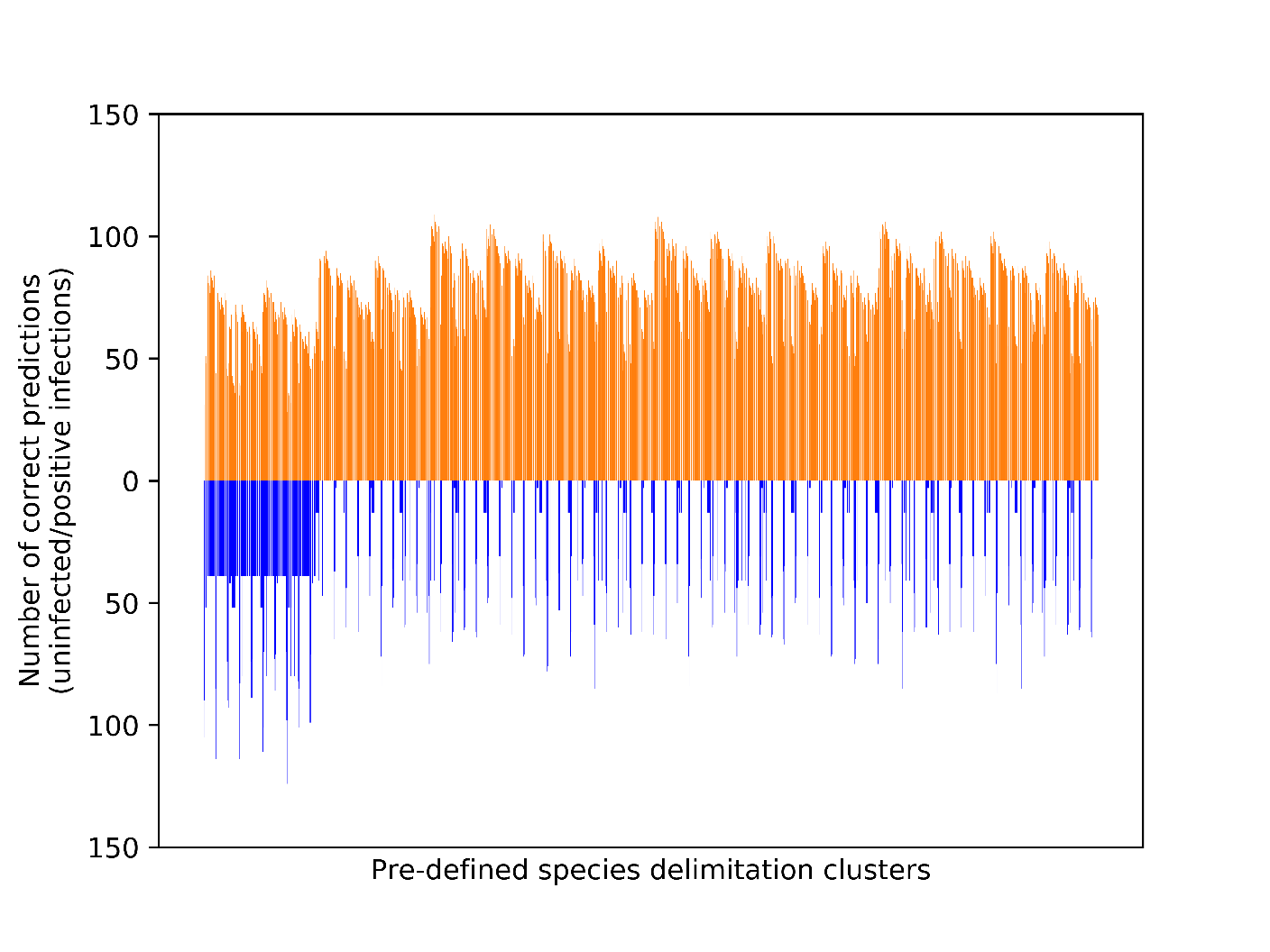


**Figure S4.** Predictive accuracy of wolPredictor across 8192 (unordered) species delimitation cluster permutations according to population elevations within wasp communities and among all communities. Results also indicate predictions accuracies for 10 purging increments per species delimitation cluster. Above the zero-axis indicates correct positive strain predictions; below the zero-axis (blue) indicates correct uninfected predictions. It is noticeable that correct positive strain predictions trades-off against correct uninfected prediction accuracies. For positive strains a maximum of 109/119 (91.60%) correct predictions were made.


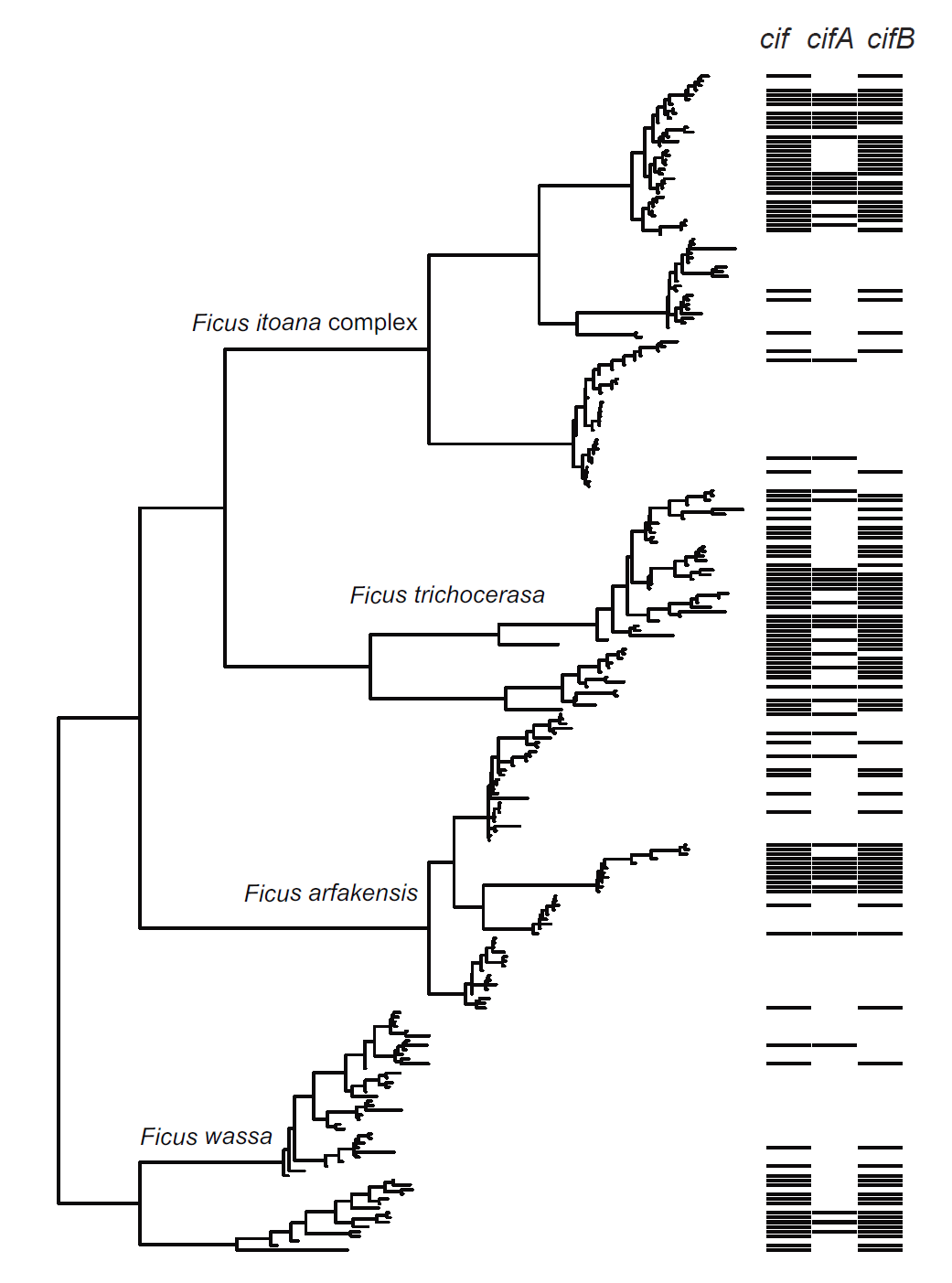


**Figure S5**. A Bayesian molecular phylogeny of wasps derived from unfiltered next-RAD data (please see the main text) with presence (black) and absence (white) of i) *cif*, ii) *cifA* and iii) *cifB* indicated.

**Table S1**. Table of Genbank accession used as Blast queries for identifying *cif* genes.

| Code and Accession Number | Taxon, Host and Region |  |  |  |  |  |
| --- | --- | --- | --- | --- | --- | --- |
| A_D.mel_1 ENA\|QQL96217\|QQL96217.1 | Wolbachia endosymbiont of Drosophila melanogaster cytoplasmic incompatibility factor CifA | | | | | |
| A_D.mel_2 ENA\|QQL97361\|QQL97361.1 | Wolbachia endosymbiont of Drosophila melanogaster cytoplasmic incompatibility factor CifA | | | | | |
| A_D.mel_3 ENA\|QQL98491\|QQL98491.1 | Wolbachia endosymbiont of Drosophila melanogaster cytoplasmic incompatibility factor CifA | | | | | |
| A_D.mel_4 ENA\|QQL99715\|QQL99715.1 | Wolbachia endosymbiont of Drosophila melanogaster cytoplasmic incompatibility factor CifA | | | | | |
| A_D.mel_5 ENA\|QQM00850\|QQM00850.1 | Wolbachia endosymbiont of Drosophila melanogaster cytoplasmic incompatibility factor CifA | | | | | |
| A_C.sol ENA\|QTP63008\|QTP63008.1 | Wolbachia endosymbiont of Ceratosolen solmsi cytoplasmic incompatibility factor CifA | | | | | |
| A_D.tei ENA\|TLW85490\|TLW85490.1 | Wolbachia endosymbiont of Drosophila teissieri cytoplasmic incompatibility factor CifA | | | | | |
| A_D.san ENA\|TLW86988\|TLW86988.1 | Wolbachia endosymbiont of Drosophila santomea cytoplasmic incompatibility factor CifA | | | | | |
| A_D.yak ENA\|TLW88872\|TLW88872.1 | Wolbachia endosymbiont of Drosophila yakuba cytoplasmic incompatibility factor CifA | | | | | |
| B_D.mel_1 ENA\|QQL96218\|QQL96218.1 | Wolbachia endosymbiont of Drosophila melanogaster cytoplasmic incompatibility factor CifB | | | | | |
| B_D.mel_2 ENA\|QQL97945\|QQL97945.1 | Wolbachia endosymbiont of Drosophila melanogaster cytoplasmic incompatibility factor CifB | | | | | |
| B_D.mel_3 ENA\|QQL99077\|QQL99077.1 | Wolbachia endosymbiont of Drosophila melanogaster cytoplasmic incompatibility factor CifB | | | | | |
| B_D.mel_4 ENA\|QQM00308\|QQM00308.1 | Wolbachia endosymbiont of Drosophila melanogaster cytoplasmic incompatibility factor CifB | | | | | |
| B_D.mel_5 ENA\|QQM01442\|QQM01442.1 | Wolbachia endosymbiont of Drosophila melanogaster cytoplasmic incompatibility factor CifB | | | | | |
| B_C.sol ENA\|QTP63507\|QTP63507.1 | Wolbachia endosymbiont of Ceratosolen solmsi cytoplasmic incompatibility factor CifB | | | | | |
| B_W.pip ENA\|QED01727\|QED01727.1 | Wolbachia pipientis cytoplasmic incompatibility factor CifB | | | |  |  |

**Table S2**. A table of closest match alleles in the MLST data base for each strain and gene. Taxa are selected to be representative of their strain. Numbers correspond to allele number in the MLST data base and phylogenies above (e.g. SnoWas27 is a representative of the wspC1 clade, the allele that is closest in the MLST data base for its hcpa sequence is 80). Note that wspC6 and wspC7 share the same nearest match allele for wsp.

| Taxon | Strain | Allele *wsp* | Allele *hcpa* | Allele *gatB* | Allele *ftsZ* | Allele *fbpA* | Allele *coxA* |
| --- | --- | --- | --- | --- | --- | --- | --- |
| SnoWas27 | wspC1 | 18 | 80 | 7 | 229 | 130 | 244 |
| DegArf58 | wspC2 | 663 | 258 | 88 | 229 | 147 | 192 |
| NumArf40R | wspC3 | 75 | 213 | 66 | 229 | 147 | 32 |
| MemTri9R | wspC5 | 11 | 60 | 179 | 52 | 17 | 15 |
| SnoTri6R | wspC6 | 81 | 128 | 261 | 52 | 17 | 221 |
| SnoMic046 | wspC7 | 81 | 210 | 58 | 52 | 442 | 32 |

**Table S3a.** Breakdown of recorded *cifA* reads across wasp species clades. ‘N stop regions’ indicates where alleles are non-orthologous but overlap on region of gene (i.e., start positions overlap).

| Species | n | Tot.  reads | Unique  reads | Samples with stops | Tot. stop reads | N unique stop reads | N stop orthologs | N stop regions |
| --- | --- | --- | --- | --- | --- | --- | --- | --- |
| arfakensis 1 | 11 | 803 | 17 | 1 | 176 | 3 | 1 | 1 |
| arfakensis 2 | 9 | 1 | 1 | 0 | 0 | 0 | 0 | 0 |
| arfakensis 3 | 28 | 19 | 2 | 0 | 0 | 0 | 0 | 0 |
| arfakensis 4 | 16 | 0 | 0 | 0 | 0 | 0 | 0 | 0 |
| umbrae | 32 | 2 | 2 | 0 | 0 | 0 | 0 | 0 |
| itoana | 22 | 0 | 0 | 0 | 0 | 0 | 0 | 0 |
| microdictya | 35 | 85 | 9 | 1 | 1 | 1 | 1 | 1 |
| pleioclada 1 | 34 | 537 | 16 | 2 | 21 | 2 | 2 | 2 |
| pleioclada 2 | 14 | 46 | 6 | 0 | 0 | 0 | 0 | 0 |
| wassa 1 | 16 | 963 | 18 | 2 | 96 | 7 | 3 | 2 |
| wassa 2 | 36 | 28 | 3 | 1 | 9 | 1 | 2 | 1 |

**Table S3b.** Breakdown of recorded *cifB* reads across wasp species clades. ‘N stop regions’ indicates where alleles are non-orthologous but overlap on region of gene (i.e., start positions overlap).

| Species | n | | Tot.  reads | Unique  reads | Samples with stops | Tot. stop reads | N unique stop reads | N stop orthologs | N stop  regions |
| --- | --- | --- | --- | --- | --- | --- | --- | --- | --- |
| arfakensis 1 | | 11 | 18687 | 275 | 7 | 1576 | 36 | 8 | 4 |
| arfakensis 2 | | 9 | 7 | 2 | 0 | 0 | 0 | 0 | 0 |
| arfakensis 3 | | 28 | 16 | 5 | 0 | 0 | 0 | 0 | 0 |
| arfakensis 4 | | 16 | 26 | 4 | 0 | 0 | 0 | 0 | 0 |
| umbrae | | 32 | 15 | 5 | 0 | 0 | 0 | 0 | 0 |
| itoana | | 22 | 28 | 3 | 0 | 0 | 0 | 0 | 0 |
| microdictya | | 35 | 2834 | 68 | 1 | 6 | 1 | 1 | 1 |
| pleioclada 1 | | 34 | 1577 | 47 | 1 | 6 | 1 | 1 | 1 |
| pleioclada 2 | | 14 | 1444 | 29 | 1 | 6 | 1 | 1 | 1 |
| wassa 1 | | 16 | 9667 | 138 | 3 | 42 | 7 | 3 | 3 |
| wassa 2 | | 36 | 28 | 5 | 0 | 0 | 0 | 0 | 0 |

**Table S4.** Table of uninfected *wsp*/MLST wasps displaying positive *cif* associations (n = 36). Most cases are found among wasp clades that have predominantly positive *wsp*/MLST associations which generally improves within clade fixation levels. It is likely that for such tiny wasp samples (and subsequent DNA extraction volumes), Sanger sequencing of *wsp*/MLST may have sometimes proved ineffective. As well as *cif*-*wsp*/MLST incongruence, there is also mismatch between *cifA* and *cifB* detection levels (again, presumably due to stochastic sequencing performance). ○ indicates functional *cif* read identification while ● indicates reads found with stop codons present.

| Wasp code | Host fig species | Clade | *cifA* | *cifB* |
| --- | --- | --- | --- | --- |
| DegArf52 | *arfakensis* | subsp.1 | ○ | ● |
| DegArf52R | *arfakensis* | subsp.1 | ○ | ○ |
| DegArf59R | *arfakensis* | subsp.1 | ○ | ○ |
| DegArf60R | *arfakensis* | subsp.1 | ○ | ● |
| DegAfa051 | *arfakensis* | subsp.1 |  | ○ |
| MemArf7 | *arfakensis* | subsp.2 | ○ | ○ |
| MemAfr001 | *arfakensis* | subsp.2 | - | ○ |
| KauArf012b | *arfakensis* | subsp.3 | - | ○ |
| NumArf006 | *arfakensis* | subsp.4 | - | ○ |
| DegImi1Rc | *umbrae* | - | ○ | - |
| DegImi6R | *umbrae* | - | ○ | - |
| DegImi001 | *umbrae* | - | - | ○ |
| DegImi7R | *umbrae* | - | - | ○ |
| BruMic050 | *microdictya* | - | ○ | ○ |
| SnoMic53R | *microdictya* | - | ● | ○ |
| BruMic049 | *microdictya* | - | - | ○ |
| BruMic36R | *microdictya* | - | - | ○ |
| BruMic49bc | *microdictya* | - | - | ○ |
| DegTri21 | *pleioclada* | *pleioclada* | ● | ○ |
| SnoTri11b | *pleioclada* | *pleioclada* | ○ | - |
| DegTri024 | *pleioclada* | *pleioclada* | - | ○ |
| DegTri027 | *pleioclada* | *pleioclada* | - | ○ |
| DegTri17c | *pleioclada* | *pleioclada* | - | ○ |
| DegTri23 | *pleioclada* | *pleioclada* | - | ○ |
| DegTri23b | *pleioclada* | *pleioclada* | - | ○ |
| DegTri25c | *pleioclada* | *pleioclada* | - | ○ |
| DegTri27b | *pleioclada* | *pleioclada* | - | ○ |
| SnoTri7c | *pleioclada* | *pleioclada* | - | ○ |
| DegWas71 | *wassa* | subsp.1 | - | ● |
| SnoWas022 | *wassa* | subsp.1 | - | ● |
| SnoWas17 | *wassa* | subsp.1 | - | ○ |
| KauWas031 | *wassa* | subsp.2 | ● | - |
| KauWas029 | *wassa* | subsp.2 | - | ○ |
| NumWas028 | *wassa* | subsp.2 | - | ○ |
| NumWas031 | *wassa* | subsp.2 | - | ○ |
| NumWas31R | *wassa* | subsp.2 | - | ○ |

**Appendix**

*Field Site Details*

All samples were collected along a continuously forested transect spanning 2,500m in elevation located on the Northern slopes of New Guinea’s Central range. The lowest site, Kausi, was situated at 200 m.a.sl. (latitude: 5.73, longitude: 145.33) and sampling camps were established every 500 m in elevation until the highest camp, Bruno Sawmill, at 2,700 m.a.sl. (latitude: 5.82, longitude: 145.15). Kausi is situated in lowland forest in the river Ramu flood-plain while the remaining sites are situated within low to mid-elevation montane forest. A nearby weather station in Usino received an average annual 3,488 mm of precipitation between 2011 and 2016 and this is 3,454 mm at the Mt Wilhelm weather station close to our highest site. Mean average temperature over the same period was 25.3ºC at Usino and 10.8ºC at Mt Wilhelm. There is an almost permanent layer of cloud between our two highest sites.

Detailed metadata (including co-ordinates and elevation of each sample) can be found in the electronic appendix (‘*wolPredictorSI_metaData.xlsx’*), while a map of the sampling sites is shown below (Appendix figure 1). Transects were selected to cover a wide range of local habitat, forming part of a wider survey of *Ficus* species outlined in Segar et al. (2017). Briefly, we identified 12,880 individuals from 73 species of fig in our wider surveys, this is around 45% of the country's 157 *Ficus* species. We found the dissimilarity in *Ficus* communities to increase with elevation while species richness peaked at 700 m.a.s.l. and declined with elevation up until 2,700 m.a.s.l. (no figs are found above this elevation). In some cases, species were locally rare or did not occur in our transects, this meant wider searches were needed. Most notably, *F. wassa* was sampled in an adjacent valley rather than at our main site at 1,200 m.a.s.l..

**
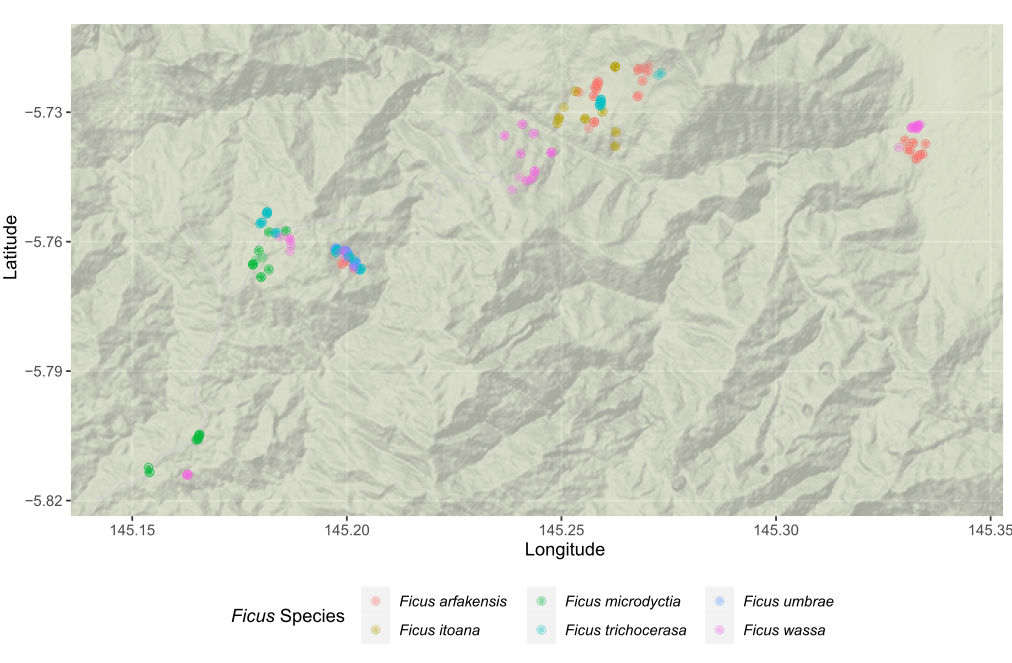
**

**Appendix figure 1.** A map of sampling locations for each tree located along the Mt Wilhelm elevational transect spanning from lowland floodplain forest to mid-elevation montane forest.

**Fig Biology**

Our study species were selected according to overall local abundance and to represent a range of genetic diversity: from widespread species found across elevations to a monophyletic species complex with parapatric replacement. Our study includes monoecious and dioecious species as well as one that is gynomonoecious (the only know representative in the genus). Most of the species here can be found growing as shrubs or trees (10-20 m), apart from *F. trichocerasa* which can be up to 25 m tall (Berg and Corner, 2005).

**Wasp Biology**

The wasps associated with our focal species remain understudied, the most up to date molecular treatment was by Souto-Vilaros et al. (2019). No sharing of wasps was found among different species of *Ficus* host, including the species complex comprising *F. itoana*, *F. umbrae* and *F. microdyctia* and the subspecies of *F. trichocerasa*. However, two species of *Ceratosolen* pollinator were associated with *F. arfakensis* at 200 m.a.s.l. and 700 m.a.s.l. (neither restricted to a single elevation) while an individual from one of these species was found in figs collected at 1,200 m.as.l. In general populations of *F. arfakensis* at 1,200 m.a.s.l. and 1,700 m.a.s.l were pollinated by specific wasp species. It is harder to strictly delimit the wasps associated with *F. wassa*, but there is a clear clade associated with all fig individuals up to and including 1,200 m.a.s.l. while a distinct and separate clade pollinates trees from 1,700 m.a.s.l. to 2,700 m.a.s.l. more detailed analysis of co-ancestry suggests further splitting of wasps associated with *F. wassa* along the transect (Souto-Vilaros et al., 2019 Figure 3) but this is partly based on mtDNA. Wasps typical of lowland host populations were occasionally found at higher elevation populations of the same host as shown in Figure 3 as presented in Souto-Vilaros et al. (2019).

**References**

Segar, S.T., Volf, M., Zima Jnr, J., Isua, B., Sisol, M., Sam, L., *et al.* (2017). Speciation in a keystone plant genus is driven by elevation: a case study in New Guinean *Ficus*. *Journal of Evolutionary Biology*, 30, 512–523.

Souto-Vilarós, D., Machac, A., Michalek, J., Darwell, C.T., Sisol, M., Kuyaiva, T., *et al.* (2019). Faster speciation of fig-wasps than their host figs leads to decoupled speciation dynamics: Snapshots across the speciation continuum. *Mol. Ecol.*, 28, 3958–3976.

Souto-Vilarós, D., Proffit, M., Buatois, B., Rindos, M., Sisol, M., Kuyaiva, T., *et al.* (2018). Pollination along an elevational gradient mediated both by floral scent and pollinator compatibility in the fig and fig-wasp mutualism. *J. Ecol*.
